# Refining the pH response in *A* *spergillus nidulans*: a modulatory triad involving PacX, a novel zinc binuclear cluster protein

**DOI:** 10.1111/mmi.13173

**Published:** 2015-10-16

**Authors:** Henk‐Jan Bussink, Elaine M. Bignell, Tatiana Múnera‐Huertas, Daniel Lucena‐Agell, Claudio Scazzocchio, Eduardo A. Espeso, Margherita Bertuzzi, Joanna Rudnicka, Susana Negrete‐Urtasun, Maria M. Peñas‐Parilla, Lynne Rainbow, Miguel Á. Peñalva, Herbert N. Arst, Joan Tilburn

**Affiliations:** ^1^ Section of MicrobiologyImperial College LondonFlowers Building, Armstrong RoadLondonSW7 2AZUK; ^2^ Manchester Fungal Infection GroupInstitute for Inflammation and RepairUniversity of Manchester46 Grafton StreetManchesterM13 9NTUK; ^3^ Department of Cellular and Molecular BiologyCentro de Investigaciones Biológicas CSICRamiro de Maeztu 9Madrid28040Spain; ^4^ Institute for Integrative Biology of the Cell (I2BC)CEACNRSUniversité Paris‐SudOrsayFrance

## Abstract

The *A* *spergillus nidulans* PacC transcription factor mediates gene regulation in response to alkaline ambient pH which, signalled by the Pal pathway, results in the processing of PacC^72^ to PacC^27^ via PacC^53^. Here we investigate two levels at which the pH regulatory system is transcriptionally moderated by pH and identify and characterise a new component of the pH regulatory machinery, PacX. Transcript level analysis and overexpression studies demonstrate that repression of acid‐expressed *pal* *F*, specifying the Pal pathway arrestin, probably by PacC^27^ and/or PacC^53^, prevents an escalating alkaline pH response. Transcript analyses using a reporter and constitutively expressed *pac* *C* *trans*‐alleles show that *pac* *C* preferential alkaline‐expression results from derepression by depletion of the acid‐prevalent PacC^72^ form. We additionally show that *pac* *C* repression requires PacX. *pac* *X* mutations suppress PacC processing recalcitrant mutations, in part, through derepressed PacC levels resulting in traces of PacC^27^ formed by pH‐independent proteolysis. *pac* *X* was cloned by *impala* transposon mutagenesis. PacX, with homologues within the Leotiomyceta, has an unusual structure with an amino‐terminal coiled‐coil and a carboxy‐terminal zinc binuclear cluster. *pacX* mutations indicate the importance of these regions. One mutation, an unprecedented finding in *A* *. nidulans* genetics, resulted from an insertion of an endogenous *Fot1*‐like transposon.

## Introduction

Fungi, ubiquitous in nature, occupy niches of wide ranging and fluctuating pH values. This is enabled by efficient pH homeostasis and a pH regulatory system that ensures the appropriate synthesis of molecules with respect to environmental pH. The system mediating this response was first recognised in *Aspergillus nidulans* (Caddick *et al*., 1986). Homologous systems occur throughout the ascomycetes where they are known as the Pac/Pal system in the filamentous fungi (Caddick *et al*., 1986; Tilburn *et al*., 1995) and as the Rim system in yeasts (Su and Mitchell, 1993a, 1993b; Lambert *et al*., 1997; Ramon *et al*., 1999; Davis *et al*., 2000a, 2000b). They also extend to the basidiomycetes (Aréchiga‐Carvajal and Ruiz‐Herrera, 2005; O'Meara *et al*., 2010; Ost *et al*., 2015).

The fungal pH responsive regulatory domain encompasses a very large number of genes including those involved in nutrient acquisition, ion homeostasis, alkali metal and pH tolerance, cell wall metabolism, exported metabolite production, female development, sporulation, dimorphic shift, tissue penetration and invasive growth (Lamb *et al*., 2001; Lamb and Mitchell, 2003; Bensen *et al*., 2004; Eisendle *et al*., 2004; Baek *et al*., 2006; Ruiz and Ariño, 2007; Nobile *et al*., 2008; Alkan *et al*., 2013; Trushina *et al*., 2013; Bertuzzi *et al*., 2014; Chinnici *et al*., 2014; O'Meara *et al*., 2014). As many of these activities or attributes are crucial in a host environment, pH regulation is an important virulence determinant of fungal pathogenicity of animals, including humans, plants and fungi themselves (Davis *et al*., 2000a; Davis, 2003; Bignell *et al*., 2005; Moreno‐Mateos *et al*., 2007; Nobile *et al*., 2008; Zou *et al*., 2010; Alkan *et al*., 2013; Trushina *et al*., 2013; Bertuzzi *et al*., 2014; O'Meara *et al*., 2014) and reviewed in Peñalva *et al*. (2008), Davis (2009), Selvig and Alspaugh (2011) and Cornet and Gaillardin (2014).

The mechanism of pH regulation has been studied largely in *A. nidulans* and *Saccharomyces cerevisiae* with additional contributions particularly from work in *Candida albicans* and *Yarrowia lipolytica*. The pH response is mediated by the three Cys2‐His2 finger transcription factor, *A. nidulans* PacC (Tilburn *et al*., 1995) or Rim101 in *S. cerevisiae* (Su and Mitchell, 1993b). Under acidic conditions, the *A. nidulans* PacC full‐length form, PacC^72^, is protease inaccessible due to intramolecular‐interactions involving the C‐terminal moiety (Espeso *et al*., 2000). At neutral to alkaline ambient pH PacC undergoes two‐step proteolysis. The first step, which occurs in response to pH signalling, removes approximately 180 C‐terminal residues to yield PacC^53^ (Díez *et al*., 2002); the second, almost certainly mediated by the proteasome, removes a further ∼ 240 residues to give the PacC^27^ processed form and is pH‐independent (Hervás‐Aguilar *et al*., 2007). PacC^27^ predominates in the nucleus (Mingot *et al*., 2001), where it activates alkaline‐expressed genes, such as *ipnA* (isopenicillin‐N synthase) (Espeso and Peñalva, 1996) and represses acid‐expressed genes (Tilburn *et al*., 1995), such as *gabA* (GABA permease) (Espeso and Arst, 2000). However, PacC^72^ and PacC^53^ can bind a PacC DNA target site (Díez *et al*., 2002) and, as they are not excluded from the nucleus (Mingot *et al*., 2001; Davis, 2003; Fernández‐Martínez, A Hervás‐Aguilar MAP, EAE, unpublished), might also participate in gene regulation.

*pacC* mutations that remove the PacC^72^ C‐terminus or otherwise disrupt its intramolecular‐interactions result in an open, proteasome‐accessible conformation leading to constitutive PacC processing and alkalinity mimicry (Orejas *et al*., 1995; Tilburn *et al*., 1995; Espeso *et al*., 2000). Loss‐of‐function mutations in the *pal* (pH signal transduction) genes or *pacC* result in acidity mimicry (Arst *et al*., 1994; Tilburn *et al*., 1995; Fernández‐Martínez *et al*., 2003). It appears that traces of PacC*^27^* are produced independently of pH signal transduction from a minor proportion of protease accessible PacC*^72^* that exists in equilibrium with the majority of protease inaccessible PacC^72^ (Peñalva and Arst, 2004; Peñas *et al*., 2007; Peñalva *et al*., 2008). This possibly explains the less extreme phenotype of null *pal* compared with *null pacC* mutations, which result, additionally, in cryosensitivity and reduced growth and conidiation (Tilburn *et al*., 1995; Fernández‐Martínez *et al*., 2003).

pH signalling occurs at the plasma membrane in *A. nidulans* (Galindo *et al*., 2012; Lucena‐Agell *et al*., 2015) and *S. cerevisiae* (Obara and Kihara, 2014) where it involves dedicated Pal (Arst *et al*., 1994) (or Rim) pH signal transduction components and the participation of certain endosomal sorting complex required for transport (ESCRT)‐I, ‐II and ‐III components (Xu *et al*., 2004; Calcagno‐Pizarelli *et al*., 2011; reviewed by Peñalva *et al*., 2014). The plasma membrane sensor is PalH [Rim21 and Dgf16 in *S. cerevisiae* and *C. albicans* (Barwell *et al*., 2005; Rothfels *et al*., 2005)]. PalH localisation is assisted by PalI (Calcagno‐Pizarelli *et al*., 2007) and stabilised by strong interactions between the PalH cytoplasmic tail and the arrestin PalF (Herranz *et al*., 2005) (Rim8). PalF becomes phosphorylated and ubiquitylated in alkaline media (Herranz *et al*., 2005), and the importance of this ubiquitylation, in *A. nidulans*, is demonstrated by the ability of genetically encoded ubiquitin attachment to PalF to signal constitutively (Hervás‐Aguilar *et al*., 2010). PalF recruits Vps23 of ESCRT‐I (Herrador *et al*., 2010; Galindo *et al*., 2012), which is thought to recruit ESCRTII components. Vps32 of ESCRT‐III participates in the incorporation of PalC (Galindo *et al*., 2007) [probably YGR122w in yeast and named YlRim23 in *Y. lipolytica* (Blanchin‐Roland, 2011)], which is required for inclusion of PalA (Galindo *et al*., 2012) (Rim20). PalA binds PacC^72^ via two YPxL(I) motifs flanking the signalling protease (PalB) cleavage site (Vincent *et al*., 2003). Finally the cysteine protease PalB (Denison *et al*., 1995) (Rim13) is recruited through interaction of its MIT domain with Vps24 (Rodríguez‐Galán *et al*., 2009; Lucena‐Agell *et al*., 2015). The transient signalling foci are dissociated by Vps4 (Galindo *et al*., 2012).

Mutations in *pacC* that prevent PacC signalling proteolysis affect the signalling proteolysis site (Díez *et al*., 2002; Peñas *et al*., 2007) or PalA binding sites (Vincent *et al*., 2003) and are phenotypically identical to those in the *pal* signal transduction genes except that, unlike *pal* ^−^ mutations that are recessive, they are co‐dominant with the wild‐type allele in diploids (see below).

The Pac/Pal system mediates a rapid and effective response to alkalinisation, switching genes on or off as appropriate to ensure survival in these adverse conditions. However, fungi also grow in acidic environments and normally prefer to do so. *A. nidulans* can grow in media of pH values as low as pH 2 (Dijkema *et al*., 1986) and in a study of the influence of pH on the growth of toxigenic *Aspergillus*, *Penicillium* and *Fusarium* species, the majority of the 61 isolates were able to grow around pH 3–3.5 and some as low as pH ∼ 2 (Wheeler *et al*., 1991). To adapt to acidic conditions, fungi must be able to control, even switch off, the alkaline ambient pH response. Here we describe, for *A. nidulans*, a tripartite system whereby this is achieved that includes a new player in the pH response.

## Results

### New insights into the *pac* *C* */pal* pH regulatory response

#### *pac* *C* autoregulation, revisited

*pacC* is an alkaline expressed gene. In wild‐type strains *pacC* transcript levels are low under acidic conditions and relatively high under alkaline conditions (Tilburn *et al*., 1995). In addition, they are low in acidity mimicking *pal* ^−^ and certain *pacC* ^+/−^ partial loss‐of‐function strains and relatively high in *pacC* ^c^ constitutive, alkalinity mimicking mutants, irrespective of the growth pH (Tilburn *et al*., 1995). This strongly suggested that *pacC* is positively regulated by PacC^27^, in a similar manner to alkaline expressed structural genes, such as *ipnA* (Orejas *et al*., 1995; Tilburn *et al*., 1995; Espeso and Peñalva, 1996).

However, the hypothesis of *pacC* positive autoregulation failed to explain a number of subsequently observed phenomena. First, in contrast to *pal* ^−^ alleles that are recessive to the respective wild‐type alleles in diploids, *pacC* processing recalcitrant alleles *pacC* ^+/−^20205, *pacC* ^+/−^207, *pacC* ^+/−^209 and *pacC* ^+/−^210 are co‐dominant with the wild‐type allele in diploids, a feature that enabled the isolation of *pacC* ^+/−^207, *pacC* ^+/−^209 and *pacC* ^+/−^210 as suppressors for GABA utilisation in a homozygous *areA* ^r^ (unable to use nitrogen sources other than ammonium) diploid (Arst *et al*., 1994; Espeso and Arst, 2000; Díez *et al*., 2002; Fernández‐Martínez *et al*., 2003; Vincent *et al*., 2003). Second, and in agreement, there are reduced levels of expression of *pacC* ^c^700, a GFP tagged allele encoding PacC^27^, in a diploid containing *pacC* ^+/−^209 as compared with that of a *pacC*700 haploid strain or of *pacC*700 in a diploid strain containing a *pacC* null allele, as detected by epifluorescence microscopy (Fig. S1A). Third, there exists an apparently paradoxical phenomenon whereby the hypostasis of a *pal* ^−^ allele and the epistasis of a *pacC* ^c^ allele can be inverted by ectopic overexpression of *pacC* ^+^ from a strong heterologous [*alcA* (alcohol dehydrogenase)] promoter in a *pacC* ^c^ *pal* ^−^ strain (Fig. S1B and JT, J Mingot, M Orejas, T Suárez, EAE, MAP, HNA, unpublished). All of these observations indicate that there is a negative function or activity associated with the PacC unprocessed form.

**Figure 1:**
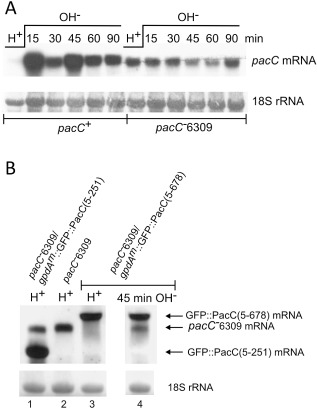
*pac* *C* is an alkaline‐expressed gene due to derepression resulting from depletion of PacC^72^ rather than activation by PacC^27^ upon alkalinisation. A and B. Northern blots of total RNA probed with P ^32^‐(A)and DIG‐ (B) labelled *pac* *C* specific probes prepared with primers TILREV and 1217FF (A) and 850U and 1217FF (B) and methylene blue stained rRNA as loading controls. The mycelia were grown overnight in acidic medium and transferred to alkaline medium for the times indicated. ‘Drop out’ medium and MFA were used in (A) and (B) respectively. *pac* *C* ^−^6309 is a null allele that can specify only residues 1–4 (Fernández‐Martínez *et al*., 2003). The wild‐type *pac* *C* allele used in (A) was *pac* *C*900, which encodes a 3× MYC tag at the amino‐terminus (Peñas *et al*., 2007), hence the reduced mobility of the transcripts relative to those of *pac* *C* ^−^6309, which differ from wild type in only a few nucleotides. Comparing the 90 min time points for the two strains suggests that *pac* *C* ^−^6309 transcript levels are not fully derepressed; however, this might be due to reduced transcript stability of *pac* *C* ^−^6309 mRNA, for instance, through non‐sense‐mediated decay. (B) *pac* *C* ^−^6309 transcript is used as a reporter of *pac* *C* gene expression to observe the effects of PacC^72^ [*gpdA* ^mini^::GFP::PacC5‐678 in acidic medium (H ^+^)] and PacC^27^ (*gpdA* ^mini^::GFP::PacC5‐251) on *pac* *C* gene expression. The *gpd* *A* ^mini^::GFP::PacC strains contain the *trans* genes at *pyro* *A* *. gpd* *A* ^mini^ is a moderate strength, constitutive promoter derived from the glyceraldehyde 3‐phosphate dehydrogenase (*gpd* *A*) promoter as described by Pantazopoulou and Peñalva (2009). Strains J2402 *paba* *A*1 *pyro* *A*4 *pac* *C* ^−^6309, J2422 and J2427 (*Experimental procedures*) were used.

The testing of the original hypothesis directly was enabled by the isolation of the *pacC* ^−^6309 null allele, which differs from the wild‐type transcript in only three nucleotides and contains a chain termination mutation in the physiological start codon, such that it can specify at most PacC residues 1 through 4 by using an alternative start codon (Fernández‐Martínez *et al*., 2003). The northern blots in Fig. 1A show the results of pH shift experiments. In the wild‐type strain *pacC* transcript levels are low under acidic conditions and rapidly rise upon alkalinisation to peak approximately 15 min after shifting, falling to steady‐state levels after about 60 min. In contrast, in the *pacC* ^−^6309 null strain, *pacC* transcript levels are largely constant throughout and considerably elevated relative to those of the wild‐type strain under acidic conditions. As PacC^72^ is the almost exclusive PacC form in acidic media, this strongly implicates PacC^72^ as a repressor of *pacC*.

**Figure 2:**
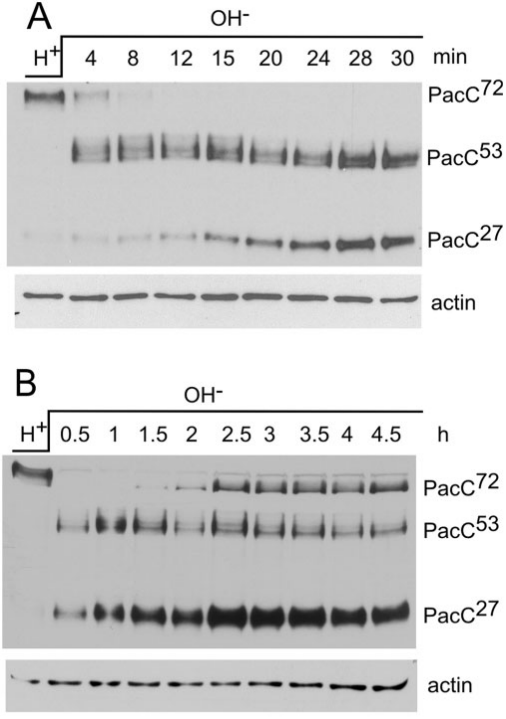
Western blots illustrating PacC processing. (A) pH signalling proteolysis occurs rapidly after alkalinisation and, (B) becomes rate limiting in PacC processing after 1.5–2 h. (A) and (B) Mycelia were grown in acidic media and transferred to alkaline media for the times shown. A *pac* *C*900 [MYC3‐PacC, (Peñas *et al*., 2007)] strain MAD2352 *w* *A*4 *pyro* *A*4 *ino* *B*2 *pal* *F*500 *pyr* *G*89 *nku* *A*Δ::bar *pac* *C*900 was used. [*pal* *F*500 is *pal* *F*::HA3::*pyr* *G* *fum*, a *pal* *F* ^+^ tagged allele (Hervás‐Aguilar *et al*., 2010).

To investigate the possibility of PacC^72^ repressor function further we used *pacC* ^−^6309 transcript as a reporter for *pacC* expression in the presence and absence of different *pacC* alleles in *trans*, expressed from a moderately strong constitutive promoter. Fig. 1B shows that *pacC* ^−^6309 transcript levels (lane 2) are unaffected by the expression of GFP::PacC5‐251, which approximates PacC^27^, (lane 1, merodiploid) but are undetectable in the presence of GFP::PacC5‐678 under acidic conditions where PacC is almost exclusively in the PacC^72^ form (lane 3). In the same strain, after 45 min of exposure to alkaline medium, which results in very extensive processing of PacC^72^ (see Fig. 2), *pacC* ^−^6309 transcripts are restored to appreciable levels (lane 4). These results give further compelling evidence that PacC^72^ is a repressor of *pacC* expression. Furthermore, PacC^27^, originally hypothesised to be an activator of *pacC* expression (Orejas *et al*., 1995; Tilburn *et al*., 1995), appears not to have an effect.

These data strongly suggest that *pacC* is negatively autoregulated by PacC^72^ and is alkaline‐expressed due to derepression, which occurs upon PacC^72^ processing in response to pH signalling. Thus, *pacC* processing recalcitrant alleles are negatively *trans*‐acting in diploids with wild‐type or constitutive alleles because their gene product represses the heteroallelic promoter (Fig. S1A). In a similar way, in a haploid, a *pal* ^−^ allele becomes epistatic to a *pacC* ^c^ allele when *pacC* ^+^ is ectopically overexpressed due to large amounts of PacC^72^ switching off expression of the *pal*‐independent *pacC* ^c^ allele (Fig. S1B).

#### Transcriptional regulation of *pal* *F* prevents a run‐away alkaline pH response

**Figure 3:**
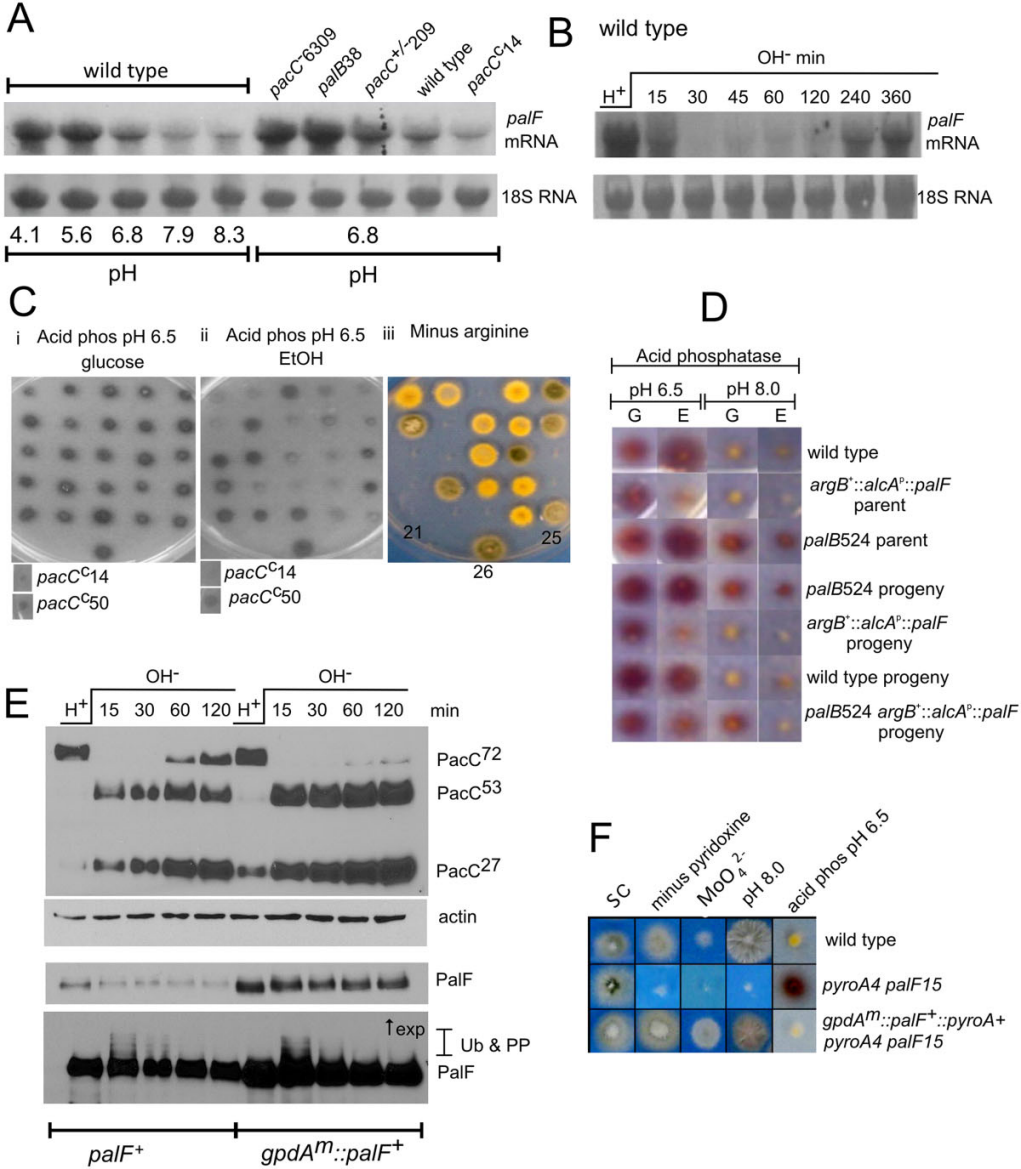
*palF* is an acid expressed gene and its overexpression results in alkalinity mimicry. A and B. Northern blots of total RNA probed with P ^32^ labelled *pal* *F*‐specific probe. Methylene blue stained rRNA is a loading control. Strains were grown in MFA‐derived media either in steady‐state conditions at the pH values shown (A) or shifted from pH 4.3 (H ^+^) to pH 8.3 (OH ^−^) for up to 6 h (B). Northerns show that *pal* *F* is preferentially expressed under acidic growth conditions and in the acidity mimicking strains carrying *pac* *C* ^−^6309, *pal* *B*38 and *pac* *C* ^+/−^209 as compared with in the wild type and the alkalinity mimicking *pac* *C* *^c^*14strains (A) and that *pal* *F* transcript levels quickly fall on shifting from acidic to alkaline growth conditions (B). C. Progeny from a cross of an *arg* *B*2 (arginine requiring) strain carrying *pac* *C*900 [MYC_3_‐tagged *pac* *C* ^+^ (Peñas *et al*., 2007)] [*paba* *A*1 *y* *A*2 *pyr* *G*89 *arg* *B*2 *pac* *C*900 (MP12)] and a strain carrying *alc* *A* ^p^::*pal* *F*::*arg* *B* ^+^ containing the *pal* *F* overexpressing cassette driven by the alcohol‐inducible alcohol dehydrogenase promoter and integrated at *arg* *B* to restore arginine prototrophy [*alc* *A* ^p^::*pal* *F*::*arg* *B* ^+^ *panto* *B*100 (JR110)] are shown. Position 21 *arg* *B*2 *pac* *C*900 (MP12), position 25 *alc* *A* ^p^::*pal* *F*::*arg* *B* ^+^(JR110), position 26 *bi* *A*1 wild‐type strain. (Ci) acid phosphatase stain (Acid phos) on minus phosphate medium containing 1% glucose pH 6.5, (Cii) acid phosphatase stain on minus phosphate medium containing 1% ethanol as carbon source pH 6.5 and (Ciii) synthetic complete glucose medium minus arginine. *pac* *C* ^c^14 and *pac* *C* ^c^50, which are strong and moderate *pac* *C* constitutive alleles, respectively, are shown below the acid phosphatase plates. Comparison of panels ii and iii demonstrates co‐segregation of arginine prototrophy and reduced acid phosphatase levels under *alc* *A* ^p^ inducing (*pal* *F* overexpression) conditions. D. Suppression of *palB*524 by *pal* *F* overexpression. Acid phosphatase stained colonies are shown after growth on minus phosphate medium containing 1% glucose (G) or 1% ethanol (E). Repressed levels of acid phosphatase are restored in the *pal* *B*524 *arg* *B* ^+^::*alc* *A* ^p^::*pal* *F* progeny after growth on ethanol. *palB*524 is a leaky loss of function allele described by Peñas *et al*. (2007). The genotype of the *pal* *B*524 *arg* *B* ^+^::*alc* *A* ^p^::*pal* *F* progeny was checked by sequencing to confirm the *pal* *B*524 mutation and Southern blot analysis to confirm the presence of the overexpression cassette. E. Western blot comparing PalF levels and PacC processing in shifted strains containing endogenously or constitutively (*gpdA* ^mini^) expressed *pal* *F* shows that elevated PalF levels correlate with increased PacC processing and that attenuation of the pH signal, manifest by accumulation of PacC^72^ with time after shifting, is almost completely removed when *pal* *F* is overexpressed from the heterologous (*gpd* *A* ^mini^) promoter. Strains MAD2352 and MAD4500 were used. F. Petri dish assays show the alkalinity mimicking phenotype of *gpd* *A* ^mini^::GFP::PalF::*pyroA* ^+^ *palF*15 (TM280) (labelled as *gpdA^m^*::*palF* ^+^::*pyroA* ^+^ *pyroA*4 *palF*15) as compared with a wild‐type (*bi* *A*1) strain with respect to molybdate resistance and reduced acid phosphatase staining. The *pyro* *A*4 *pal* *F*15 recipient strain (TM261) is also shown. The full genotypes of the strains are described in *Experimental procedures*.

Figure 2 illustrates PacC processing. In response to alkalinisation, PacC^72^ is processed via PacC^53^ to PacC^27^. The response is very swift with PacC^53^ appearing after 4 min (Panel A) and, in fact, as early as 2.5 min (data not shown). After 30–60 min PacC^72^ has disappeared yet between 90 and 150 min PacC^72^ begins to accumulate again, indicating that PacC processing has become limited at the signalling proteolysis step (Fig. 2B). Therefore, the possibility of pH regulation of transcription of the pH regulatory *pal* genes and the consequences of their overexpression were explored. *palA*, ‐*B*, ‐*C*, ‐*H* and ‐*I* were found to be expressed largely independently of pH and/or the mutational status of other pH regulatory components, i.e., *pacC* and *palF* (Denison *et al*., 1995; 1998; Negrete‐Urtasun *et al*., 1997; 1999). However, *palF* was found to be an acid expressed gene (Fig. 3A and B). *palF* transcript levels are highest under acidic conditions and in acidity mimicking mutants and relatively low under alkaline growth conditions and in alkalinity mimicking mutants (Fig. 3A). Moreover, on shifting from acidity to alkalinity, *palF* transcript levels rapidly fall very low between 30 and 120 min after transfer, being somewhat restored after 4 h (Fig. 3B). This transcriptional behaviour resembles the temporal pattern of PacC processing and suggests that expression of *palF* might be rate‐limiting in pH signal transduction. This is supported by overexpression studies (Fig. 3C and D) that show that *palF* expression from the strong, inducible *alcA* (alcohol dehydrogenase) promoter results in alkalinity mimicry, as indicated by reduced acid phosphatase expression on ethanol‐containing, low phosphate medium, whereas there were no such phenotypic consequences of the overexpression of *palA*, ‐*B*, ‐*C*, ‐*H* or ‐*I* (data not shown). In addition, *palF* overexpression is sufficient to suppress the very leaky *palB*524 mutation (Peñas *et al*., 2007), for acid phosphatase expression (Fig. 3D) and to rescue partially certain leaky mutations in *palH* and *palC* and *palI*32 (data not shown). [*palI*32 is a null allele which, like all *palI* mutations, has a less extreme phenotype than non‐leaky mutations in the other *pal* signal transduction pathway genes (Denison *et al*., 1998)]. *palF* overexpression from a modified *gpdA* (glyceraldehyde 3‐phosphate dehydrogenase) promoter (*gpdA* ^mini^) (Pantazopoulou and Peñalva, 2009) also results in alkalinity mimicry as detected in Petri dish assays (Fig. 3F and Hervás‐Aguilar *et al*., 2010). Western blot analysis demonstrates that *gpdA* ^mini^ driven *palF* overexpression results in elevated levels of PacC processing and largely overrides attenuation of pH signalling as manifested by the much reduced levels of PacC^72^ detectable 60 min and 120 min after shifting (Fig. 3E). The appreciable levels of PacC^27^ produced under acidic growth conditions (Fig. 3E) are indicative of pH signalling and suggest that, in agreement with the alkalinity mimicking phenotype, constitutive expression of PalF results in an inappropriate pH response.

These results suggest that the *A. nidulans* pH regulatory response is subject to negative feedback in a fashion similar to that involving *RIM8* of *C. albicans* and *S. cerevisiae* (Porta *et al*., 1999; Ramon *et al*., 1999; Lamb and Mitchell, 2003). These results also support some recent mathematical modelling predictions that suggest the presence of a negative feedback loop in the PacC activation process (Ke *et al*., 2013).

### *pac* *X*: a new gene involved in the pH regulatory response

#### Identification and genetic mapping of *pac* *X*

The first identified *pacX* mutation, designated *pacX*1, was isolated, following UV mutagenesis of a *pacC* ^+/−^20205 (processing recalcitrant) strain, among numerous (largely intragenic) revertants selected for their ability to grow at pH 8.0. *pacX*1, that segregated as a single trait, independently of *pacC*, was localised further to chromosome VIII using parasexual genetics. In view of this localisation and the *pacX*1 phenotype, which includes the partial restoration of alkaline phosphatase biosynthesis, it seemed possible that *pacX*1 is allelic to *suA*1*palB*7, a mutation linked to the *argC*3 translocation breakpoint on chromosome VIII, isolated and characterised by Dorn (1965), during his studies of the phosphatases of *A. nidulans*. Guided by these results, and those of Clutterbuck (1993), *pacX*1 was mapped 8.3 and 16 map units respectively between the *argC*3 translocation breakpoint and *hisC*38. Allelism with *suA*1*palB*7 and *suD*2*palA*1, also isolated by Dorn (http://fgsc.net/Archive/nid.html), was confirmed by further genetic analysis (data not shown) and determination of mutant sequence changes (see below).

### Characterisation of the *pac* *X* mutant phenotype: by‐passing pH signal transduction

**Figure 4:**
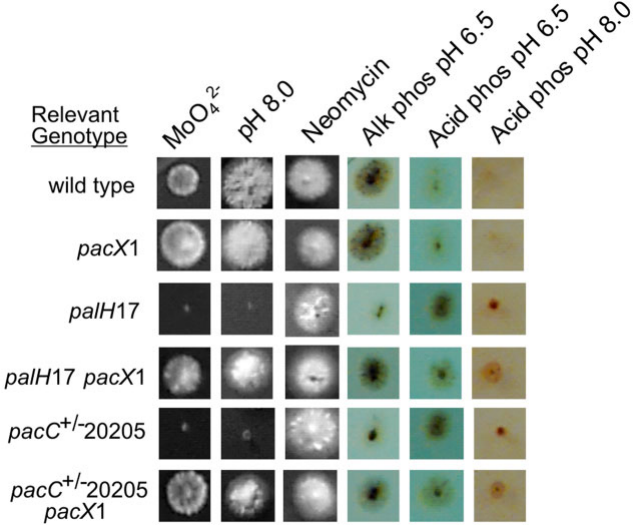
Suppression of *pal* ^−^ and *pac* *C* ^+/−^20205 mutations by *pac* *X*1. Petri dish assays of typical strains following 48 h growth on synthetic complete medium containing 25 mM sodium molybdate (MoO_4_^=^), 1 mg ml^−1^ neomycin sulphate (Neomycin), pH 8.0 medium (pH 8.0) and phosphatase staining following 24 h growth on minus phosphate medium at the pH values shown, followed by staining for alkaline or acid phosphatase (Alk phos or Acid phos), are shown.

*pacX*1 was found to suppress loss‐of‐function mutations in each of the *pal* pH signal transduction genes, specifically, *palA*1, *palB*7, *palC*4, *palF*15, *palH*17 and *palI*30. Phenotype testing of the *pacX*1 *palA* ^−^, ‐*B* ^−^, ‐*C* ^−^, ‐*F* ^−^, ‐*H* ^−^ and ‐*I* ^−^, *pacX*1 *pacC^+/^* ^−^20205 (Fig. 4), *pacX*1 *pacC* ^+/−^207, *pacX*1 *pacC* ^+/−^209 and *pacX*1 *pacC* ^+/−^210 (data not shown) double mutants showed that, in addition to partial restoration of growth at pH 8.0 and alkaline phosphatase biosynthesis, *pacX*1 reduces the derepressed levels of acid phosphatase to levels intermediate between those of the *pal* ^−^, *pacC^+/^* ^−^20205, *pacC* ^+/−^207, *pacC* ^+/−^209 and *pacC* ^+/−^210 strains and those of wild‐type strains and *pacX*1 not only abolishes molybdate sensitivity, it results in moderate molybdate resistance relative to wild‐type strains and similar to that observed in *pacC* ^c^ constitutive strains. *pacX*1 single mutants are alkalinity mimicking, molybdate resistant and have somewhat elevated levels of alkaline phosphatase and reduced levels of acid phosphatase (hence the *pac* designation) (Fig. 4). In addition, they have slightly reduced conidiation at pH 6.5 and they are resistant to lithium toxicity. Thus, in Petri dish tests, *pacX*1 single mutants resemble weak *pacC* ^c^ constitutive mutants.

#### The range of *pac* *C* alleles phenotypically modified by *pac* *X* ^−^ mutations

Numerous *pacX* mutations have been selected as suppressors of *pal* ^−^ or *pacC* ^+/−^ processing recalcitrant mutations (Table 1). All extant *pacX* mutations have the same phenotype and *pacX* mutant sequence changes clearly show that these are loss‐of‐function (see, for *example*, *pacX*3503 and *pacX*20, Table 1). The range of *pacC* alleles affected by *pacX* ^−^ mutations was further explored by phenotype testing of a variety of *pacC* alleles in combination with *pacX* ^−^ mutations. The results (Table 2) demonstrate that *pacX* ^−^ mutations are unable to suppress *pacC* ^−^ null mutations and severe *pacC* truncation alleles. However, *pacX* ^−^ mutations enhance the toxicity of the overexpressed *alcA* ^p^::MYC::PacC6‐253 allele, which approximates the PacC processed form. In addition, *pacX*1 is additive with the weak constitutive mutations *pacC* ^c^234, *pacC* ^c^39 and *pacC* ^c^20042; that is, the *pacC* ^c^ *pacX*1 double mutants are more alkalinity mimicking than the *pacC* ^c^ single mutants (Table 2 and data not shown). The epistasis of *pacC* ^−^ alleles to *pacX* ^−^ alleles strongly suggests that PacC acts downstream of, or is more directly involved than PacX in the pH regulatory response.

**Table 1:** *pac* *X* mutations isolated in this work

Allele	Nucleotide change	Protein change	Mutagen	Selection	Reference
Large insertions or duplication					
*pacX*12^a^	T‐516*ins* *impala*	PPI	None	Growth at pH 7.5	This work
*pacX*18^a^	T667*ins* *Fot1*‐like transposon	PSD	None	Growth at pH 8.0	This work
*pacX*24^b^	T‐297*ins*(C‐296‐T878)	N275fs, PPI	None	Growth at pH 8.0	This work
Truncating mutations					
*pacX*3503^i^	A35*ins*G25‐A37	G12fs	None	Growth at pH 8.0	This work
*pacX*20^a^	C133T	R44stop	None	Growth at pH 8.0	This work
*pacX*28^a^	C180GG	P60fs	None	Growth at pH 7.5	This work
*pacX*8^a^	ΔC190	I63fs	UV	Growth at pH 8.0	This work
*pacX*13^a^	ΔG(241‐243)	G81fs	None	Growth at pH 8.0	This work
*pacX*26^a^	G314‐316*ins*G	R105fs	None	Growth at pH 8.0	This work
*pacX*34^c^	G331T	H110stop	None	Growth at pH 8.0	This work
*pacX*19^b^	(T359‐G364)A	W199fs	None	Growth at pH 8.0	This work
*pacX*33^a^	A379‐A385i*ns*A	K127fs	None	Growth at pH 7.5	This work
*pacX*3501^i^	ΔA445‐A449	Y148fs	None	Growth at pH 8.0	This work
*pacX*3502^i^	ΔC505	M168fs	None	Growth at pH 8.0	This work
*pacX*27^a^	G554T	D181stop	None	Growth at pH 7.5	This work
*pacX*31^a^	C687T	R211stop	None	Growth at pH 7.5	This work
*pacX*4^d^	(C942‐944)*ins*C	P297fs	NQO	Growth at pH 8.0	Denison and Arst, unpublished
*pacX*2^e^ (*su*A1*pal*B7)	ΔG945	G299fs	None	Alkaline phosphatase	(Dorn, 1965)
*pacX*15^a^	(G945‐951)*ins*G	G299fs	None	Growth at pH 8.0	This work
*pacX*3511^i^	(G945‐951)*ins*G	G299fs	None	Growth at pH 8.0	This work
*pacX*3506^i^	A951*ins*GGAGAAG	G299GKfs	None	Growth at pH 8.0	This work
*pacX*1^a^	ΔC957, ΔC958	A301fs	UV	Growth at pH 8.0	This work
*pacX*3512^i^	ΔT(108‐1090)	F345fs	None	Growth at pH 8.0	This work
*pacX*3513^i^	G1128*ins*C1121‐G1128	Y347fs	None	Growth at pH 8.0	This work
*pacX*5^d^	ΔG1250	A399fs	NQO	Growth at pH 8.0	Denison and Arst, unpublished
*pacX*6^d^	ΔCG (1340‐1345)	A430fs	NQO	Growth at pH 8.0	Denison and Arst, unpublished
*pacX*25^a^	ΔT1391	D246fs	None	Growth at pH 8.0	This work
*pacX*14^a^	ΔG1393	R447fs	None	Growth at pH 8.0	This work
*pacX*32^a^	ΔC(1526‐1529)	P492fs	None	Growth at pH 7.5	This work
*pacX*3508^i^	ΔC(1526‐1529)	P492fs	None	Growth at pH 8.0	This work
*pacX*3509^i^	G1530insA	P492Sfs	None	Growth at pH 8.0	This work
*pacX*3507^i^	ΔC1571, ΔA1572	S506fs	None	Growth at pH 8.0	This work
Missense mutations					
*pacX*3^f^ (*suD*2*palA*1)	C130T	R44W (ef)	None	Alkaline phosphatase	Dorn*
*pacX*16^a^	T248C	L83P (bs)	None	Growth at pH 8.0	This work
*pacX*21^a^	C310A	P104T (ef)	None	Growth at pH 8.0	This work
*pacX*23^a^	G357T	W119C (bs)	None	Growth at pH 8.0	This work
*pacX*17^a^	ΔG458‐G460	(R153, (ef) V154L) (bs)	None	Growth at pH 8.0	This work
*pacX*3505^i^	G679C	R209P (ef)	None	Growth at pH 8.0	This work
*pacX*22^a^	G700C	R216P (ef)	None	Growth at pH 8.0	This work
*pacX*9^g^	G715C	R221P (ef)	NQO	Growth at pH 8.0	Denison and Arst, unpublished
*pacX*11^h^	C721G	A223G (ef)	None	None	This work
*pacX*7^a^	C1384T	P444L (ef)	None	Molybdate resistance	This work
*pacX*30^a^	G1396T	C448F (bs)	None	Growth at pH 7.5	This work
*pacX*29^a^	G1468T	C472F (bs)	None	Growth at pH 7.5	This work
*pacX*10^h^	P1470C	S473P (ef)	UV	Molybdate resistance	Akintade and Tilburn, unpublished

Mutation isolated as suppressor of: ^a^ *pacC* ^+/−^20205, (Díez *et al*., 2002), ^b^ *pacC* ^+/−^ 207 (Vincent *et al*., 2003), ^c^ *palF*58 (Arst *et al*., 1994), ^d^ *palI*30 (Denison *et al*., 1998), ^e^ *palB*7 (Dorn, 1965; Peñas *et al*., 2007),^f^ *palA*1 (Dorn, 1965), ^g^ *palI*49 (Arst *et al*., 1994; Denison *et al*., 1998), ^h^ *pacC*209 (Díez *et al*., 2002), ^i^ *pacC*900^L498S^ (Peñas *et al*., 2007). ^i^mutations were isolated in *pacX*35 which is an S‐tagged *pacX* ^+^ allele (PacX::4GA::S‐tag, see *Experimental procedures*). PPI, predicted promoter insertion; PSD, predicted splicing defect. The ConSurf (http://consurf.tau.ac.il/) predictions for substituted residues are given in parentheses: ef, exposed and functional; bs, buried and structural. See *Experimental procedures* for media. *pacX*11 arose spontaneously in strain *pacC* ^+/−^209 *pantoB*100 (L186) on storage. Dorn*, Dorn http://fgsc.net/Archive/nid.html.

**Table 2:** Modified and unmodified *pac* *C* allele phenotypes

*pacC* allele	Phenotype	PacC mutant protein (unprocessed form)	Effect of *pacX* ^−^ in double mutant
Modified phenotype			
*pacC* ^+/−^207^b^	Acidity mimicry	Y455N	Suppression
*pacC* ^+/−^209^b^	Acidity mimicry	L498S	Suppression
*pacC* ^+/−^210^b^	Acidity mimicry	L498F	Suppression
*pacC* ^+/−^20205^b^ ^,^ *	Acidity mimicry	5‐464 IDRPGSPL 541‐678	Suppression
*pacC* ^c^39^b^	Weak alkalinity mimicry	L266F	Additivity
*pacC* ^c^234^b^	Weak alkalinity mimicry	L340F	Additivity
*pacC* ^c^20042^b^	Weak alkalinity mimicry	R579T	Additivity
*pacC* ^+/−^20002^b^	Acidity mimicry	Q155K	Enhanced loss‐of‐function phenotype
*pacC* ^c/−^20000^c^	Neutrality mimicry	5‐251 + 5	Additivity
*alcA* ^p^::MYC::PacC6‐253^a^	Alkalinity mimicry	5‐252	Increased toxicity
No modification of phenotype			
*pacC* ^−^Δ::*Ncpyr4* ^a^	Null		
*pacC* ^−^6310^c^	Null	5‐163	
*pacC* ^+/−^7604^b^	Acidity mimicry	5‐173 + 9	
*pacC* ^+/−^230^c^	Acidity mimicry	5‐238	
*pacC* ^*c*^700 *pacC* ^+/−^70001^c^	Acidity mimicry	GFP::5‐250 K159M	
*pacC* ^c/−^20601^c^	Neutrality mimicry	5‐260	
*pacC* ^c^50^c^	Alkalinity mimicry	5‐266	
*pacC* ^+/−^206^c^	Acidity mimicry	5‐310	
*pacC* ^c^69^b^	Alkalinity mimicry	L340S	
*pacC* ^+/−^7601^c^	Acidity mimicry	5‐379	
*pacC* ^c^504, *pacC*5^b^	Weak neutrality mimicking	1‐523	

The *pacX* allele is indicated after the *pacC* allele number: ^a^ *pacX*20, ^b^ *pacX*1, ^c^ *pacX*3.*The *pacC* ^+/−^20205 allele also includes *pacC* ^c^202. Allele *alcA^p^*::MYC::PacC6‐253 specifies M‐MYC(EQKLISEEDL)‐AAAS‐PacC6‐253 and is integrated in *pyroA* in an otherwise *pacC* null background.

Although it appears that a *pacX* ^−^ phenotypic manifestation requires the PacC processed form to be largely functional, not all conforming alleles are affected. For example, the alkalinity mimicry of *pacC* ^c/−^20000 (5‐251 + 5) is enhanced, with respect to morphology, by *pacX* ^−^, but *pacC* ^c/−^20601 (5‐260) (Mingot *et al*., 1999), which contains the intact processed form and has a similar phenotype, appears to be unaffected. *pacC* ^c^69 (L340S) and *pacC* ^c^50 (5‐266) (Tilburn *et al*., 1995; Mingot *et al*., 1999; Espeso *et al*., 2000) do not appear to be phenotypically enhanced by *pacX*1, possibly because increased alkalinity mimicry in these strong constitutive backgrounds requires sensitivity beyond that of Petri dish tests. Lack of suppression of acidity mimicking mutants *pacC* ^+/−^230 (PacC5‐238fs) and *pacC* ^+/−^206 (PacC5‐310fs) (Mingot *et al*., 1999), which are both suppressed by mutations affecting the proteasomal degradative pathway (JT and HNA, unpublished), suggests that the effects of *pacX*1 are insufficient to offset this process. Possibly for similar reasons, *pacC* ^+/−^7601 (5‐379fs) (Tilburn *et al*., 1995), which contains a long frameshifted tail, is also unaffected. *pacC*504 (*pacC*5) M5I (5‐523) (Tilburn *et al*., 1995), is a muted *pacC* ^c^5 allele due to mutation removing the preferred translation start site and lack of additivity (data not shown) suggests that *pacX*1 is insufficient to compensate for this. Hypostasis (data not shown) of *pacX*3 to *pacC*700 *pacC* ^+/−^70001 that prevents PacC^27^ nuclear import (Fernández‐Martínez *et al*., 2003) implies that suppression by *pacX*3 requires appropriate localisation of PacC^27^.

#### Molecular effects of *pac* *X* ^−^ mutations

**Figure 5:**
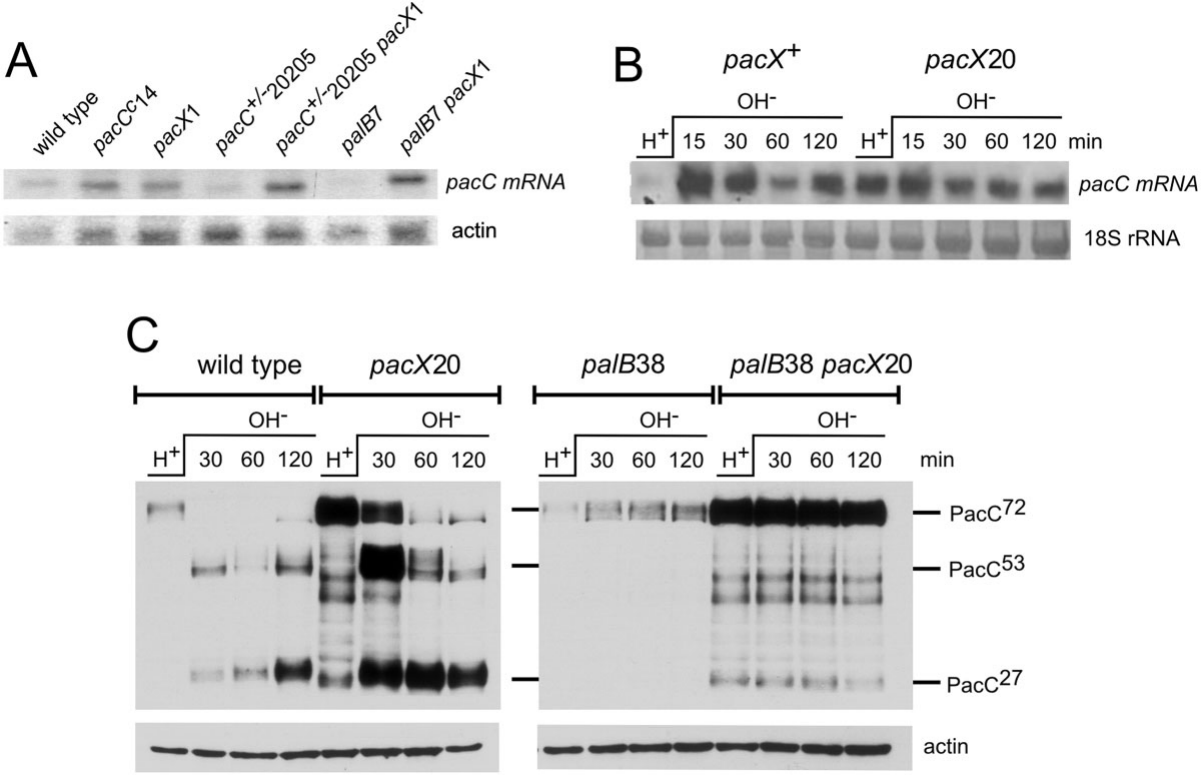
*pacX* ^−^ mutations result in derepressed expression of *pac* *C*. (A and B) Northern blots of total RNA are shown. A. The *pac* C transcript was detected among RNA from a variety of strains grown at neutral pH, pH ∼ 6.5, using a ^32^ P‐labelled 1041 bp *pac* *C*‐specific fragment, which had been generated by PCR using the primers BIGFF and TILREV (Table S1). Mycelia were grown for 14 h in shaken minimal medium, containing 1% glucose as sole carbon source and 10 mM 2‐(*N*‐morpholino)ethanesulphonic acid, at 30°C. Loading controls were established using a 650 bp *Nco*I‐*Nco*I fragment from the *A* *spergillus nidulans* actin gene (Fidel *et al*., 1988). B. Strains were grown overnight (∼ 14 h) at 37°C with shaking in acidic MFA and transferred to alkaline MFA for the times shown. Methylene blue stained 18S rRNAs are included as loading controls. *pac* *X* ^+^, *y* *A*2 *paba* *A*1 *pac* *C*900 (J2153) and *pac* *X*20, *yA*2 *paba* *A*1 *pac* *C*900 *pac* *X*20 (X900A) strains were used. C. Western blots of cell lysates. The 3× MYC tagged *pac* *C* ^+^ allele (*pac* *C*900) was carried by all strains (Peñas *et al*., 2007). The strains were pre‐grown in acidic media and transferred to alkaline media for the times indicated. *pacX* ^+^, *pyro* *A*4 *pac* *C*900 (MAD3877); *pac* *X*20, *y* *A*2 *paba* *A*1 *pac* *X*20 *pac* *C*900 (MAD1652); *pal* *B*38, *ino* *B*2 *pal* *B*38 *pac* *C*900 (MAD1362) and *pal* *B*38 *pac* *X*20, *pac* *C*900 *panto* *B*100 *pac* *X*20 *pal* *B*38 (MAD4777) strains were used.

*pacX* ^−^ mutations result in derepression of *pacC* expression. Northern blots in Fig. 5A demonstrate that, after growth in neutral media, *pacX*1, *pacC* ^+/−^20205 *pacX*1 and *palB*7 *pacX*1 strains all have elevated *pacC* transcript levels, relative to the respective *pacX* ^+^ strains, similar to those in the *pacC* ^c^14 strain. In a pH shift experiment (Fig. 5B), the *pacX*20 strain has constant *pacC* transcript levels, independent of growth pH and similar to those obtained in the wild‐type strain in response to alkalinisation. Thus, *pacX* ^−^ mutants resemble *pacC* ^−^ null (Fig. 1A) and *pacC* ^c^ (Fig. 5A and data not shown) mutations in having derepressed *pacC* transcript levels.

The effects of *pacX*1 on *pacC* transcript levels are reflected in PacC DNA binding activity detected in EMSAs where there are considerably increased amounts of the lower mobility complex containing PacC^72^ and/or PacC^53^ from protein extracts of a *pacX*1 strain relative to those from a wild‐type strain from neutral grown mycelia (Fig. S2). The relatively modest increase in the amount of the higher mobility complex, containing PacC^27^, from the *pacX*1 strain indicates that *pacX*1 does not override the *palF* transcriptional negative feedback loop (Fig. S2A). In *pal* ^−^ or *pacC* ^+/−^20205 *pacX*1 double mutants, which are phenotypically pH independent, there are increased amounts of both the lower and higher mobility complexes relative to those in the *pal* ^−^ or *pacC* ^+/−^20205 single mutants (Fig. S2B and C).

Western blot analyses of shift experiments in Fig. 5C, where all three PacC forms can be distinguished, confirm that the increased amounts of complexes detected in EMSAs from the *pacX* ^−^ strains are due to increased amounts of protein, rather than improved binding efficiencies. In addition they demonstrate that prior to the 120 min time point, all PacC forms, where present, are considerably elevated in the *pacX*20 strain relative to those in the wild type. After 120 min PacC levels in the *pacX*20 strain have fallen and both the relative proportions and the amounts of the three PacC forms are very similar in both strains.

PacC^72^ levels are also highly elevated in the *palB*38 *pacX*20 strain relative to those in the (null) *palB*38 single mutant and similar to those obtained in the *pacX*20 single mutant under acidic conditions (Fig. 5C). On shifting to alkalinity, levels of PacC^72^ remain fairly constant in both *palB*38 strains and appreciable amounts of PacC^27^ and partial degradation products in the *palB*38 *pacX*20 strain are detectable throughout. These bands are attributable to C‐terminal, processive, Pal‐independent proteolysis of the minor proportion of PacC^72^ having an ‘open’, and proteasome accessible, conformation that exists in equilibrium with the much more numerous PacC^72^ molecules that are ‘closed’ and proteasome resistant (Espeso and Arst, 2000; Espeso *et al*., 2000; Díez *et al*., 2002; Hervás‐Aguilar *et al*., 2007; Peñas *et al*., 2007; Peñalva *et al*., 2008).

### Cloning the *pac* *X* gene by transposon‐mediated mutagenesis

Extensive attempts to rescue the *pacX*1 mutant phenotype by co‐transformation of an *argB*2 *pacX*1 *palA*1 strain with plasmid pILJ16 carrying *argB* ^+^ (Johnstone *et al*., 1985) and chromosome VIII allocated Lorist or pWE15 derived cosmids (Brody *et al*., 1991) selecting for *argB* ^+^ and testing for the *palA*1 alkaline sensitivity phenotype on pH 8.0 medium were unsuccessful. A subsequent PCR analysis suggested that *pacX* may be absent from these libraries. Transposon‐mediated mutagenesis was chosen as an alternative cloning strategy because of the ease with which a *pacX* ^−^ mutation could be selected. This took advantage of available strains carrying the modified *impala* transposon from *Fusarium oxysporum* tagged with the *yA* gene, required for green pigmentation of conidia, inserted in the promoter of the *niaD* gene thus resulting in the inability to utilise nitrate (Li Destri Nicosia *et al*., 2001). A *yAΔ*::*Ncpyr4*, *pabaA*1, *niaD*::*impala*::*yA* ^+^, *pacC* ^+/−^20205 strain was constructed, and transposition was found to occur at a frequency of ∼ 10^−5^. Conidia of this strain were spread on pH 7.5 medium with nitrate as sole nitrogen source to select simultaneously for *pacX* ^−^ mutations and restoration of the *niaD* ^+^ genotype by excision of the *impala* transposon. Out of an estimated 600 000 transposition events, one colony was obtained. The mutant (BG2) was found, by diploid analysis, to contain the insertion on linkage group VIII. This location and the phenotype of BG2 strongly suggested that the insertion had occurred in *pacX*, as subsequently confirmed (see below). *pacX* genomic and cDNAs sequences were determined as described in *Experimental procedures*.

### *Pac* *X* sequence

**Figure 6:**
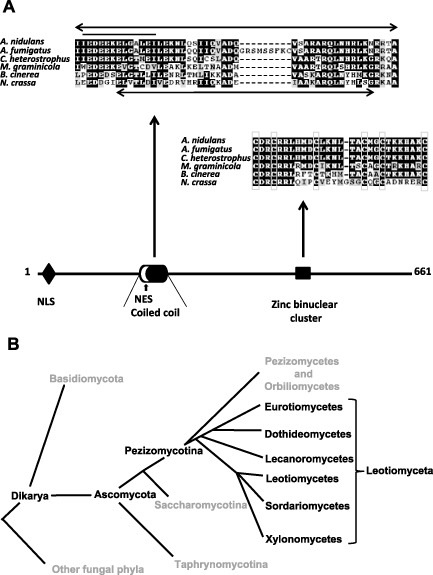
Features of PacX and its homologues. A. A scheme illustrates features of *A* *. nidulans* PacX. Portions of an alignment containing the coiled‐coil and zinc binuclear cluster regions are shown. The alignment features selected homologues with different degrees of divergence from *A* *. nidulans* PacX, *A* *spergillus fumigatus*, *C* *ochliobolus heterostrophus*, *M* *ycosphaerella graminicola* (now designated *Z* *ymoseptoria tritici*), *B* *otrytis cinerea* and *N* *eurospora crassa*. The accession numbers are shown in Fig. S6. Arrows indicate the positions of the coiled‐coil regions above the alignment for *A* *. nidulans* and below the alignment for *N* *. crassa*. The line indicates a putative nuclear export signal (NES) in the *A* *. nidulans* PacX. NLS indicates a bipartite nuclear localisation signal, which contains a shorter monopartite NLS. A putative NES is indicated within the coiled‐coil in white. Putative zinc chelating residues of the zinc binuclear cluster are indicated with boxes. B. Fungal phyla that contain PacX homologues are indicated with black lettering: those that apparently do not are indicated in grey.

The *pacX* sequence specifies a 661 residue protein. A zinc binuclear cluster DNA binding motif is located towards the carboxy‐terminus between residues 445 and 472 and a region predicted to form a coiled‐coil structure occurs in the region of residues 178 to 224 (Fig. 6 and Fig. S3). In BLAST searches of the databases, the region containing the putative DNA binding domain and the amino‐terminus extending to residue 228 are the most highly conserved (see below), indicating their functional importance (Fig. 6, Figs S3 and S4). Different algorithms all predict a nuclear localisation for PacX; ngLOC (17.6% nuclear 14.5% cytoplasmic), PSORT II (73.9% nuclear) with cNLS mapper predicting highly probable monopartite (TPGKRPRSDSGEF, residues 10 to 22, score 6.5) or bipartite (ETPGKRPRSDSGEFPPIASKVPKT, 9 to 32, score 9.0) NLSs. An NES (nuclear export sequence) is predicted with high probability by NetNES between residues 179 and 191 (Fig. 6).

The coiled‐coil prediction for *A. nidulans* PacX is 1 and therefore very strong (Fig. S3). The distancing of the coiled‐coil region from the zinc binuclear cluster is somewhat conserved among PacX homologues (Fig. 6 and Fig. S3). Coiled‐coils are putative oligomerisation domains, and the role of the coiled‐coil is supported by the partial dominance of *pacX1 vis a vis pacX^+^* (Fig. S5). *pacX*1 results in a frameshift after residue 301 (Table 2), thus conserving the coiled‐coil element but deleting the Zn cluster. A number of point mutations map within the putative coiled‐coil element (Figs S3 and S4 and Table 1).

The PacX putative DNA binding domain (Fig. 6) conforms to the consensus sequence of zinc binuclear cluster motifs (Todd and Andrianopoulos, 1997; MacPherson *et al*., 2006) characteristic of, even if not completely exclusive to, fungi (Scazzocchio, 2014). Zinc binuclear cluster DNA binding motifs are almost universally at the amino‐terminus of cognate transcription factors (MacPherson *et al*., 2006); however, that of PacX is towards the carboxy‐terminus, which resembles *S. cerevisiae* Ume6p (Strich *et al*., 1994) and *C. albicans* Czf1p (Whiteway *et al*., 1992 reviewed by MacPherson *et al*., 2006). Infrequent but not unique to PacX and its orthologues are the two Pro residues N terminal to the first Cys, and the absence of a Pro residue one or two residues N‐terminal to the fourth cysteine (Fig. S4). The dimerisation element is usually in Zn‐cluster proteins a few residues C‐terminal to the DNA binding motif, and it is typically shorter than the one seen in PacX, such as 15 residues for Gal4, 19 residues for Ppr1 (Marmorstein *et al*., 1992; Marmorstein and Harrison, 1994) with the maximal length predicted for such a C‐terminal coiled‐coil dimerisation domain being 25 residues (Schjerling and Holmberg, 1996). This contrasts with the 46 residue‐long putative coiled‐coil, 221 residues amino‐terminal to the first Cys of the Zn cluster extant in PacX. It could be proposed that while the function of the standard dimerisation domain found in Zn binuclear cluster proteins is to permit the recognition of DNA inverted, everted or direct repeats separated by a few base pairs [e.g. 11 in Gal4, 6 in Ppr1 and UaY (Marmorstein *et al*., 1992; Marmorstein and Harrison, 1994; Suarez *et al*., 1995)], the probable dimerisation of PacX may serve an altogether different function.

### Phylogenetic analysis

Searching with blastp using the PacX protein sequence as *in silico* probe of the 431 fungal genomes available in the Joint Genetics Institute database on the 30th of January 2015 showed PacX homologues to be present in the Pezizomycotina and not in any other fungal taxon, but being absent from the two sequenced genomes of the Orbiliomycetes and the seven sequenced genomes of the Pezizomycetes, basal classes of the Pezizomycotina. Absence from these and other ascomycete taxons (Saccharomycotina and Taphrynnopmycotina) was confirmed with a tblastn search. Searching the NCBI protein database, excluding the Pezizomycotina, failed to reveal any homologues with either blastp or tblastn. Although within the Eurotiomycetes the sequence is very conserved, the phylogenetic tree (Fig. S6) and the alignment (Fig. 6 and Fig. S4) demonstrate that there is much divergence within the Pezizomycotina. Within the Sordariales, the sequence even within the zinc binuclear cluster is divergent, as shown by the *Neurospora crassa* sequence in the alignment (Fig. 6). Within the Dothidiomycetes, the sequences diverge even more and form two clusters roughly corresponding to the Pleosporales and Capnodiales with one outgroup (see legend to Fig. S6 for details), secondary loss has occurred in several species, mainly within the Sordariales, including *Magnaporthe grisea* and *Podospora anserina* (confirmed by tblastn).

### Mutant sequence changes

Forty‐five mutant sequence changes confirm the identity of the gene (Table 1). Clustering of missense mutations exclusively to within the amino‐terminus and the predicted coiled coil region itself and the zinc binuclear cluster or immediately adjacent to it underscores the functional importance of these regions (Fig. S4). The results of comparing PacX with the consensus derived from an alignment of 177 PacX homologues using the ConSurf algorithms (http://consurf.tau.ac.il/) for the identification of functional regions in a protein are presented in Fig. S4, and the predictions for mutated residues are summarised in Table 1. All of the missense mutations affect conserved residues or residues that occur in conserved regions (Fig. S4). Three out of four mutations in the coiled‐coil *pacX*3505(R209P), *pacX*22(R216P) and *pacX*9(R221P) are basic to non‐polar. These are changes of Arg to Pro, a residue that breaks α‐helices. However, the coils/pcoils algorithm (http://toolkit.tuebingen.mpg.de/pcoils) only revealed in each case minor changes for the probability or length of the predicted coiled‐coil. All mutations within this region [which includes *pacX*11(A223G)] change conserved residues that are predicted to be exposed and functional and may affect a specific interaction of PacX. The majority of truncating mutations remove conserved regions of the protein. Even more extreme truncations, *pacX*3503 and *pacX*20, terminate the protein at residues 12 and 44, respectively, thus removing almost all of the protein, confirming that these classically obtained *pacX* alleles are complete loss‐of‐function (Table 1). The absence of mutations and poor conservation in the C‐terminus suggested that this region might be dispensable. This was confirmed with an engineered allele expressing PacX residues 1 to 499 from the *alcA* promoter (*alcA* ^p^::PacX1‐499) and integrated at *pyroA*, which was found to be as functional as the full‐length protein expressed from the same promoter (*alcA* ^p^::PacX1‐661) at the same integration site. *pacX*3507, which terminates the protein after Ser506 and therefore retains the functional region, has a long (41 residue) out‐of‐frame tail that might destabilise the protein or interfere with its activity and thus cause the loss‐of‐function phenotype.

*pacX* mutations also include the intergenic duplication in *pacX*24 and the *impala* transposition into the *pacX* promoter of *pacX*12. In addition, *pacX*18 was found to be an insertion identical to the *Fot1*‐like transposon in chromosome VIII defined by AN0826 (encoding the transposase). Surprisingly, sizing of the region of AN0826 amplified using external primers shows the transposon to be retained at its resident locus in the mutant strain (see Supplementary Data).

### *Pac* *X* nuclear localisation

**Figure 7:**
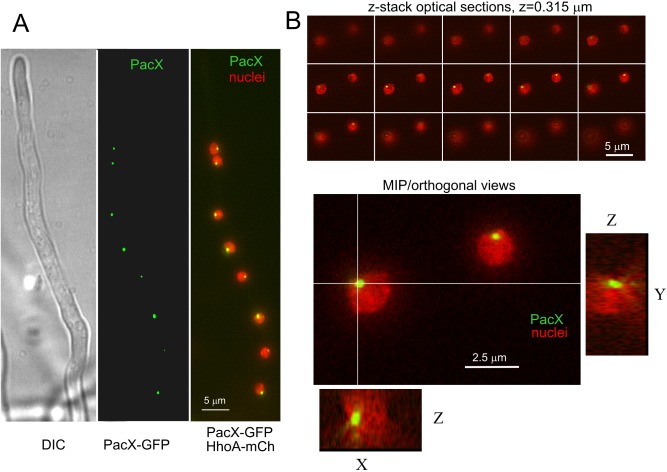
Sub‐nuclear localisation of PacX. A. Image of a hyphal tip cell coexpressing PacX‐GFP and HhoA::mCherry (histone 1) to label chromatin. DIC, Nomarski optics. B. Top, individual planes of a z‐stack of images, acquired with the indicated Z‐distance. Bottom, maximal intensity projection (MIP) of the above stack and orthogonal [(X, Z) and (Y, Z)] views across the indicated lines. All images were captured from cells cultured in acidic medium. Exposure to alkaline media for 60 min did not alter this PacX sub‐nuclear localisation pattern.

The PacX sequence, which contains putative nuclear localisation signals and a putative zinc binuclear cluster DNA binding domain, strongly suggested that PacX functions in the nucleus. PacX localisation was investigated by live epifluorescence microscopy of a strain expressing PacX::GFP and HhoA::mCherry (histone 1). Fig. 7 shows that PacX::GFP is localised within the nuclei where it forms one strongly fluorescent, discrete spot per nucleus. Shifting from acidity to alkalinity for 1 h had no effect on PacX::GFP localisation that therefore appears to be pH independent (Fig. 7). PacX localisation resembles that of the proline degradation pathway‐specific zinc binuclear cluster transcription factor PrnA, which has proline‐independent, sub‐nuclear localisation (Pokorska *et al*., 2000).

## Discussion

PacX is a protein of unusual domain structure, with a conspicuous coiled‐coil helix amino‐terminal to a typical carboxy‐terminal fungal Zn cluster. The pH signalling pathway, including the central transcription factor PacC/Rim101, is conserved throughout the ascomycetes and, to a certain extent, in the basidiomycetes and has been studied in ascomycetous yeasts and basidiomycetes (Peñalva *et al*., 2008; Davis, 2009; Selvig and Alspaugh, 2011; Blanchin‐Roland, 2013; Cornet and Gaillardin, 2014; Obara and Kihara, 2014; Peñalva *et al*., 2014; Herrador *et al*., 2015; Ost *et al*., 2015, and references therein) (in addition to *A. nidulans* and other filamentous ascomycetes). However, the additional putative transcription factor PacX is an evolutionary novelty. It occurs only in the Pezizomycotina and within the limits of the paucity of sequences available, particularly in the Orbiliomycetes, it seems to have appeared after the divergence of the other classes of the Pezizomycotina (Leotiomyceta) from the Pezizomycetes and the Orbiliomycetes. Recent studies, including those combining fossil and molecular, data differ as to whether the Pezizomycetes or the Orbiliomycetes constitute the most basal group of the Pezizomycotina (Schoch *et al*., 2009; Prieto and Wedin, 2013; Beimforde *et al*., 2014). However, these studies agree that the Leotiomyceta have diverged from the other two classes most probably in the Silurian era (∼ 430 Mya). Thus, the appearance of PacX correlates with a major phylogenetic split, which may coincide with the establishment of biotic interactions with vascular plants (Prieto and Wedin, 2013).

The characterisation of mutations in *pacX* has pin‐pointed the crucial functional regions, which correlate with those conserved throughout the Leotiomyceta. One mutation however is quite unique: In more than 60 years of *A. nidulans* genetics, this is the first and only reported mutation resulting from an insertion of an endogenous transposon. What is even more surprising is that the *Fot1* like element defined by AN0826 did not transpose by a mechanism of cut and paste, characteristic of the transposons of this class, including the heterologous transposition of the *F. oxysporum Fot1* transposon in *A. nidulans* (Li Destri Nicosia *et al*., 2001 and refs therein) but rather by a copy and paste mechanism (see Supplementary Data).

**Figure 8:**
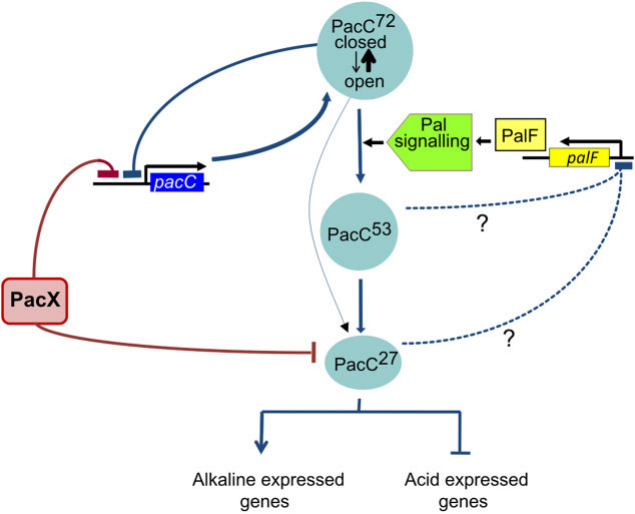
Model summarising currently available data on regulation of the pH response. The fine lines indicate that it is a minor proportion of PacC^72^ that exists in an open conformation and is therefore susceptible to Pal independent processing. The dotted lines indicate that it is unclear whether transcriptional repression of *pal* *F* is mediated by PacC^53^ or PacC^27^ and whether such repression is direct or indirect.

**Figure 9:**
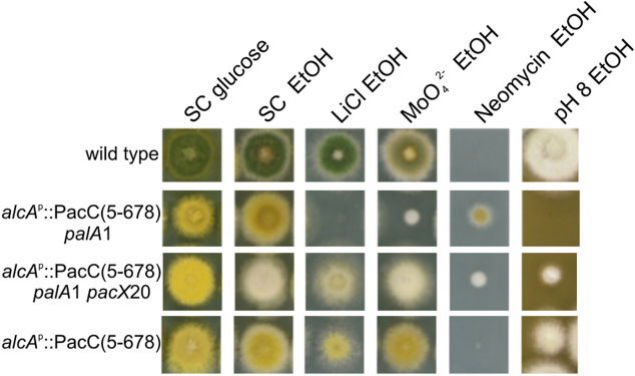
*pac* *X*20 suppresses *pal* *A*1 when *pac* *C* is expressed from a heterologous promoter. The *trans* gene expressing PacC residues 5 to 678, corresponding to the wild type allele, from the ethanol‐inducible alcohol dehydrogenase promoter, *alc* *A* ^p^::PacC5‐678 is integrated at *arg* *B* in a *pac* *C*Δ*N* *cpyr4* background (Mingot *et al*., 1999). Strains are wild‐type HB81 *panto* *B*100, *alc* *A* ^p^::PacC5‐678 *pal* *A*1 *pac* *C*Δ (9‐1), *alc* *A* ^p^::PacC5‐678 *pal* *A*1 *pac* *X*20 *pac* *C*Δ (9‐21), *alc* *A* ^p^::PacC5‐678 *pac* *C*Δ (9‐17) and full genotypes of the *trans* gene strains are given in *Experimental procedures*. Lithium chloride was added to 300 mM.

Proposed role(s) of PacX together with negative autoregulation of *pacC* and the negative feedback acting on *palF* are incorporated in a model for the control of the alkaline pH response, which is shown in Fig. 8. PacC^72^ exists predominantly in a closed proteasome inaccessible conformation in equilibrium with a small fraction of molecules assuming a less favourable open, proteasome‐accessible conformation, thus providing a substrate for the Pal‐independent bypass. In the absence of pH signal transduction, PacC^72^ is largely unprocessed (Figs 2 and 5C) and, with the participation of PacX, represses its own transcription, as demonstrated by the increased *pacC* transcript levels obtained in *pacC* null or *pacX* null strains (Figs 1A and 5B). This would reduce substrate‐driven flux through the Pal‐independent pathway. However, overexpression of *pacC* by itself produces no detectable phenotype, and overexpressing *pacC* ^+^ alleles expressed from the *alcA* or *gpdA* ^mini^ promoter are hypostatic to *pal* ^−^ mutations (Fig. 9 and Mingot *et al*., 2001). In contrast, *pacX* ^−^ mutations result in alkalinity mimicry, and they are able to suppress *pal* ^−^ and processing recalcitrant *pacC* ^+/−^ mutations. Moreover, they are able to suppress *pal* ^−^ mutations even when *pacC* is expressed from the heterologous *alcA* (Fig. 9) and *gpdA* ^mini^ (data not shown) promoters, i.e. independent of *pacC* derepression. In addition, *pacX* ^−^ mutations augment aspects of the phenotypes of *alcA* ^p^::MYC::PacC6‐253 and *pacC* ^c/−^20000 (PacC5‐251), alleles that specify truncated proteins that approximate PacC^27^ and do not require further processing (Table 2). These effects strongly suggest that PacX inhibits PacC^27^ activities in a manner separate from and in addition to its negative effects on *pacC* transcription. PacX‐mediated inhibition of PacC^27^ activities also potentially explains why the very small amounts of PacC^27^ present in *pacX* ^−^ *pal* ^−^ strains (Fig. 5C) are able to suppress the *pal* ^−^ phenotype to such an appreciable extent (Fig. 4). The alkalinity mimicking phenotype of *pacX* ^−^ mutations that result in elevated alkaline phosphatase (*palD*) and reduced acid phosphatase (*pacA*) levels demonstrates that PacX affects both activator and repressor functions of PacC^27^ and that it is required for normal responses to acidity and neutrality.

The onset of PacC processing occurs less than 4 min after exposure to alkalinity (Fig. 2A), and this rapid response would be facilitated by the pool of PalF accumulated under acidic conditions, due to *palF* being acid expressed (Fig. 3A and B). After approximately 1.5–2 h, steady‐state conditions are established and PacC^72^ begins to accumulate again (Fig. 2B), indicating that the signalling proteolysis has become limiting and that the signal has become attenuated. Work presented here, wherein overexpression of *palF* from a heterologous promoter largely prevents accumulation of PacC^72^ in alkaline media strongly suggests that repression of *palF* transcription, directly or indirectly, by PacC^53^ or PacC^27^ plays a major role in this attenuation. It appears that there is a relatively modest fall in PalF protein levels as compared with the fall in *palF* transcript levels, in response to alkalinisation. This suggests that attenuation is not a function of absolute PalF amounts but rather that of the rate of *de novo* PalF synthesis. Low rates of PalF synthesis might inhibit Pal signalling, for example, by limiting the rate of the crucial PalF ubiquitylation. Other modifications might be affected. In addition to ubiquitylation, *A. nidulans* PalF is phosphorylated in an alkaline pH dependent fashion (Herranz *et al*., 2005). Interestingly, CK1‐mediated phosphorylation of *S. cerevisiae* Rim8 has recently been reported to prevent Rim signalling (Herrador *et al*., 2015). Thus, attenuation might be achieved by limiting the rate of *de novo*, unphosphorylated, pathway‐activating PalF. An even more tempting scenario would be that the degree of signalling is a balance between the levels of ubiquitylated and phosphorylated PalF. In this situation, the effects of small changes in *de novo* synthesised PalF would be additive and could well account for the quite profound effects observed on overexpression of PalF (Fig. 3E and F).

Thus, control of the alkaline pH response ensures that both the response to alkalinisation is Pal signal transduction dependent and an escalating alkaline pH response is prevented by attenuation of pH signalling by negative feedback via repression of *palF* transcription by PacC^53^ and/or PacC^27^. It is tempting to speculate that the absence of PacX in yeasts correlates with the absence of pH signal independent activation of the pH responsive transcription factor. In *S. cerevisiae* Rim101 processing occurs in one step and is entirely Rim signal transduction dependent (Li and Mitchell, 1997; Lamb *et al*., 2001; Xu and Mitchell, 2001). In *C. albicans*, although Rim signal dependent Rim101 C‐terminal processing occurs in both acidic and alkaline media to different extents, it is thought that the alkaline active 74 kDa form is the product of a single Rim13‐mediated proteolysis (Li *et al*., 2004; Xu *et al*., 2004). However, in *Y. lipolytica*, it has been suggested that Rim101 might be processed similarly to *A. nidulans* PacC (Lambert *et al*., 1997; Blanchin‐Roland, 2013).

pH regulation of the *palF* homologues *RIM8* in *S. cerevisiae* and *C. albicans*, which are also preferentially expressed in acidic media, has been reported (Porta *et al*., 1999; Ramon *et al*., 1999; Lamb and Mitchell, 2003), suggesting that negative feedback limiting the arrestin component of the pathway might be a general feature of the pH response. However, in contrast to PalF, where ubiquitylation is strictly pH‐ and PalH‐dependent and pivotal in the response, *S. cerevisiae* Rim8 ubiquitylation appears to be pH independent and takes place in a double *rim21Δ dfg16Δ* mutant background, lacking both PalH homologues. However, the role of Rim8 ubiquitylation in Rim signalling becomes clear if the Vps23‐Rim8 interaction is debilitated by mutation of a SPX motif in the latter (Herrador *et al*., 2010). *C. albicans* Rim8 undergoes hyperphosphorylation, instead of ubiquitylation, in a manner linked to Rim101 processing (Gomez‐Raja and Davis, 2012).

Many *pacC/RIM101* homologues, like *A. nidulans pacC* [(Tilburn *et al*., 1995) and Figs 1 and 5B], are preferentially expressed under neutral to alkaline conditions. These include those of: *Beauveria bassiana* (Zhou *et al*., 2014), *C. albicans* (Bensen *et al*., 2004), *F. oxysporum* (Caracuel *et al*., 2003), *Magnaporthe oryzae* (Landraud *et al*., 2013), *Metarhizium robertsii* (Huang *et al*., 2015), *Trichoderma harzianum* (Moreno‐Mateos *et al*., 2007), *T. virens* (Trushina *et al*., 2013), *Wangiella* (*Exophial*) *dermatitidis* (Wang and Szaniszlo, 2009), *Y. lipolytica* (Lambert *et al*., 1997) but not, curiously, *S. cerevisiae* (Lamb *et al*., 2001; Serrano *et al*., 2002; Lamb and Mitchell, 2003; Viladevall *et al*., 2004). This suggests that autoregulation might be a common feature, but the absence of reporter studies precludes determining whether this would be positive or negative.

The mechanism(s) of PacX action is/are not clear but what appears to be dual functionality could be achieved by a single mechanism. For instance, if PacC^72^ and PacC^27^ were to compete for nuclear import and/or DNA binding, PacX antagonism of PacC^27^ could fulfil both functions. This might involve protein–protein interactions between PacX and PacC, possibly via the PacX coiled‐coil domain and/or direct DNA binding of PacX through the zinc binuclear cluster. PacX localisation, which is almost exclusively nuclear, supports a mechanism occurring within the nucleus.

Investigations into *pacC* and *palF* promoter occupancy, the possibility of PacC and PacX interaction, PacX sub‐nuclear localisation and post‐transcriptional regulation of PalF are topics for future research.

## Experimental procedures

### *A* *. nidulans* strains, phenotype analysis, genetic techniques and growth media

*Aspergillus nidulans* strains carried previously described markers, in general use; *s*tandard media, phenotype testing and genetic techniques were used (Caddick *et al*., 1986; Clutterbuck, 1993; Arst *et al*., 1994; Tilburn *et al*., 1995 and references therein). LiCl and MoO_4_^2−^ plates were prepared by the addition of lithium chloride or sodium molybdate solution to appropriately supplemented minimal medium (Cove, 1966) containing 1% D‐glucose and 5 mM ammonium tartrate, to the desired concentration (100–500 mM LiCl and 25 mM MoO4). Neomycin containing plates were prepared by the addition of neomycin sulphate powder to 1 or 0.5 mg ml^−1^ to molten minimal medium minus glucose, containing 1% D‐glucose or 1% ethanol (added after autoclaving), respectively, and 5 mM ammonium tartrate. pH 8.0 medium followed (Cove, 1976). Dropout media were prepared from fully supplemented yeast Dropout medium (Clontech) containing additional supplements appropriate for the auxotrophies of the strains, 5 or 10 mM urea as nitrogen source and 1% D‐glucose, added after autoclaving. Acidic dropout medium and MFA (Peñas *et al*., 2007), which contained 5 mM ammonium tartrate as nitrogen source, were buffered with 50 mM citrate to give pH 4.3, and alkaline dropout medium and MFA were buffered with 100 mM HEPES to give ∼pH 8.3. MFA was also buffered to give pH 5.6 with 100 mM NaH_2_PO_4_ (plus 100 mM NaCl); pH 6.8 with 50 mM NaH_2_PO_4_ and 50 mM Na_2_HPO_4_ (plus 50 mM NaCl) and pH 7.9 with 100 mM Na_2_HPO_4_. To obtain conidiospores for transposon mutagenesis strain 2431A (see below *Transposon mutagenesis*) was grown on minimal media (see above) with 10 mM NaNO_2_ as sole nitrogen source, buffered to pH 6.5 with 50 mM MES. To select simultaneously for growth on nitrate as sole nitrogen source and for loss‐of‐function in *pacX*, we used pH 7.5 medium, minimal medium (see above Cove, 1966) buffered to pH 7.5 by addition of 50 mM phosphate buffer (made from stock solutions 500 mM Na_2_HPO_4_ and 500 mM NaH_2_PO_4_), containing 10 mM NaNO_3_ as sole nitrogen source and 10 × PABA (40 μg ml^−1^ 4‐aminobenzoic acid).

### Construction of strains

Plasmid pALC‐*argB* (*Bgl*II) (Mingot *et al*., 1999) was used for the construction of *pacC* and *pal* overexpression cassettes containing cDNA of *pacC* and one of each of the *pal* signal transduction genes (Denison *et al*., 1995; 1998; Tilburn *et al*., 19951; Maccheroni *et al*., 1997; Negrete‐Urtasun *et al*., 1997; 1999) under *alcA* ^p^ (alcohol dehydrogenase promoter) control. This plasmid contains a functional *alcA* ^p^, containing a transcription start site, separated from the *trpC* terminator by a polylinker, and a mutant *argB* gene to direct integration to *argB* by repair of the *argB*2 allele. *pal* overexpressing strains were constructed by DNA‐mediated transformation (Tilburn *et al*., 1995) of double mutants, *argB*2 and appropriately *pal* ^−^ for the corresponding overexpressed gene, with selection for *argB* ^+^ transformants, screening for the *pal* ^+^ phenotype and Southern blot analysis to identify appropriate single copy integrants. Appropriate *pal* ^+^ transformants were subsequently crossed to obtain *pal* overexpressing strains in otherwise *pal* ^+^ genetic backgrounds. *alcA*‐driven PacC5‐678 overexpressing strains were obtained by transformation of strain MAD397 *yA*2 *argB*2 *palA*1 *pacC*Δ::*Ncpyr4 pantoB*100 with p[*alcA* ^p^::PacC5‐678], as described by Mingot *et al*. (1999), to give transformant *yA*2 *palA*1 *argB*:: *alcA* ^p^::PacC5‐678 *pacC*Δ::*Ncpyr4 pantoB*100 (MAD0415), which was crossed to HB85 *pabaA*1 (*yA*2 *or yA*Δ::*Ncpyr4*) *argB*2 *pacX*20 to give strains (9‐1) (*yA*2 or *yA*Δ*Ncpyr*4) (*pyrG*89?) *argB*::*alcA* ^p^::PacC5‐678 *palA*1 *pacC*Δ*Ncpyr4*, (9‐21) *paba*A1 (*yA*2 or *yA*Δ::*Ncpyr4*) (*pyrG*89?) *argB*::*alcA* ^p^::PacC5‐678 *palA*1 *pacC*Δ::*Ncpyr4 pacX*20 and (9‐17) *paba*A1 (*yA*2 or *yA*Δ::*Ncpyr4*) (*pyrG*89?) *alcA* ^p^::PacC5‐678 *pacC*Δ::*Ncpyr4 pantoB*100 (Fig. 9).

The *pacX* gene was 3′ tagged with GFP and S‐tag using spacer‐GFP/S‐tag‐Af*pyrG* cassettes (Yang *et al*., 2004) and introduced by gene replacement into a *nkuA*Δ::bar recipient strain (KUG4). KUG4 *pyrG*89 *pyroA*4 *niiA*4 *nkuA*Δ::bar was constructed by crossing to a strain *nkuA*Δ::bar *niiA*4 *biA*1 *pyroA*4 (kindly provided by Prof Michael Hynes). The S‐tagged *pacX* allele was denoted *pacX*35. Strain ALO2 *pacX*::GFP::AfpyrG (*pyrG*89?) *pyroA*4 HhoA::mCherry *niiA*4 *nkuA*::bar was obtained by crossing transformant A1 *pyrG*89 *pyroA*4 *niiA*4 *nkuA*Δ::bar *pacX*::GFP::Af*pyrG* with strain LO1421 containing HhoA::mCherry (kindly provided by Prof Berl Oakley).

Strains *pabaA*1 *yA*2 *gpdA* ^mini^::GFP‐PacC(5‐678)::*pyroA pacC*Δ::*Ncpyr4* (MAD1713) and *pabaA*1 *yA*2 *gpdA* ^mini^::GFP‐PacC(5‐251)::*pyroA pacC*Δ::*Ncpyr4* (MAD1710) were obtained by transformation of a *pabaA*1 *yA*2 *pacC*Δ::*Ncpyr4 pyroA*4 recipient strain with plasmids p1673 and p1666, respectively, and identification of transformed clones carrying single‐copy integration events at *pyroA* by Southern blotting. p1673 and p1666 encode GFP‐PacC(5‐678) and GFP‐PacC(5‐251) fusion proteins respectively. Coding regions were obtained as *Hin*dIII‐*Eco*RI fragments by PCR, using templates described in Mingot *et al*. (1999), and introduced into the multiple cloning site of pgpd003 (Pantazopoulou and Peñalva, 2009), downstream of the *gpdA* ^mini^ promoter. Strains J2422 *yA*2 *gpdA* ^mini^::GFP::PacC5‐678::*pyroA* ^+^ *pacC* ^−^6309 (*pacC*63) *pantoB*100 and J2427 *gpdA* ^mini^::GFP::PacC5‐251::*pyroA* ^+^ *pacC* ^−^6309 (*pacC*63) were derived by crossing MAD1713 and 1710 respectively to J2384 *areA* ^r^5 *pyroA*4 *pacC* ^−^6309 (*pacC* ^c^63) *pantoB*100 and the *pacC* ^−^6309 allele was detected among the progeny by PCR. TM280 *pabaA*1 *gpdA* ^mini^::GFP::PalF::*pyroA* ^+^ *palF*15 was obtained by transformation of recipient strain TM261 *pabaA*1 *pyroA*4 *palF*15 with pTM9015. pTM9015 was derived from p1673 by QuikChange mutagenesis (Agilent Technologies) using primers p1673‐EcoRI‐Fw and p1673‐EcoRI‐Rv (Table S1) to introduce an *Eco*R1 site into which *palF* cDNA was inserted in place of *pacC*.

The construction of endogenously expressed MYC3‐tagged PacC (allele name *pacC*900) is described by Peñas *et al*. (2007) and endogenously HA3‐tagged PalF (allele name *palF*500) or HA3‐tagged PalF expressed from *gpdA* ^mini^ promoter are described by Hervás‐Aguilar *et al*. (2010). Strain MAD2352, *wA*4 *pyroA*4 *inoB*2 *palF::HA3::pyrGfum pyrG*89 *nkuA*Δ::bar *pacC*900 was obtained by transformation of MAD1732 (Hervás‐Aguilar *et al*., 2010). MAD4500, *yA*2 *pabaA*1 *pantoB*100 *pyroA*4::[pyroA*‐*gpdA* ^mini^::*palF*::HA_3_] *pacC*900 *nkuA* ^+^ was derived by crossing MAD3319 *yA*2 *pabaA*1 *pantoB*100 and MAD3007 *pantoB*100::[pantoB*::*gpdA* ^mini^::*palH*::myc3] *pyroA*4::[pyroA*::*gpdA* ^mini^::*palF*::HA3] *pacC*900 Δ*nkuA*::*bar*?).

### Northern blots

Growth conditions for Northern blot analyses are given in the appropriate Fig. legends. RNA was extracted as described by Tilburn *et al*. (1995) or by a modified Drosophila procedure http://www.koko.gov.my/CocoaBioTech/RNA%20Isolation23.html#procedure, as follows. Lyophilised mycelium was ground in a 2 ml tube with a glass rod and mixed with 800 μl of GHCl solution (5 mM DTT, 7.5 M guanidium hydrochloride (Sigma), 25 mM sodium acetate, pH 7.0, 0.5% N‐lauryl sarcosinate) and shaken vigorously with an equal volume of phenol:chloroform:isoamylalcohol (25:24:1) or acidic (pH 4.5) phenol:chloroform:isoamylalcohol (125:24:1). After centrifugation, the RNA was precipitated from 400 μl of the aqueous phase with 16 μl of 1M acetic acid and 400 μl of ethanol with incubation at −80°C for 1–2 h. The RNA was pelleted by centrifugation and resuspended in 400 μl of GHCl solution and precipitated as before. The RNA was washed with 100% ethanol, then 75% ethanol, air dried and resuspended in RNase free water and stored at −80°C. Northern blotting followed Tilburn *et al*. (1995). Heat or UV fixed membranes were stained with methylene blue (Sambrook *et al*., 1989) and probed with appropriate radiolabelled (^32^P) or digoxygenin (DIG) labelled (Roche) DNA fragments. *pacC*‐specific probes were prepared from PCR fragments obtained with primers (Table S1) TILREV and 1217FF or BIGFF (^32^P labeled) or 850U and 1217FF (DIG labeled, Fig. 1B). Loading controls were established using a ^32^P labelled ∼ 650 bp *Nco*I‐*Nco*I fragment from the *A. nidulans* actin gene (Fidel *et al*., 1988) or methylene blue stained 18S rRNA, as indicated.

### Western blots

Mycelia were cultured and sampled essentially as described by Galindo *et al*. (2012) except that 5 mM ammonium tartrate was used as nitrogen source and alkaline medium was buffered to pH 8.0 with 100 mM HEPES. Lyophilised mycelia were homogenised, using a 5 mm ceramic bead, following Hervás‐Aguilar *et al*. (2010). Cell lysis followed a method adapted from an *S. cerevisiae* protocol (Stimpson *et al*., 2006). 6 mg samples of powdered biomass were weighed and transferred to 2 ml microcentrifuge tubes. Proteins were solubilised in 1 ml tube^−1^ lysis solution (0.2 M NaOH, 0.2% (v/v) β‐mercaptoethanol with vigorous vortexing. Proteins were precipitated with 7.5% trichloroacetic acid (TCA) and pelleted by centrifugation at 14 000 × *g* for 5 min at 4°C. Pellets were solubilised in 100 μl Tris base, mixed with 200 μl of Laemmli buffer (Laemmli, 1970) and incubated at 100°C for 2 min. Proteins (5–10 μl of each sample were resolved in 8% SDS‐polyacrylamide gel, transferred to nitrocellulose membranes. For MYC_3_::PacC and actin blots were reacted with either mouse monoclonal anti‐c‐myc (at 1/2,000 dilution) (Clone 9E10, Sigma‐Aldrich) or mouse anti‐actin monoclonal antibody (1/4,000) (Clone 4, MP Biomedicals, LLC). Peroxidase conjugated goat anti‐mouse IgG immunoglobulin (Jackson) at 1/5,000 and 1/8,000 respectively were used. PalF::HA_3_ blots were reacted and developed as described by Hervás‐Aguilar *et al*. (2010). Peroxidase activity was detected with Amersham Biosciences ECL.

### Transposon mutagenesis

*pacX* was cloned by tagging it with the *impala* transposon form *F. oxysporum* in a manner similar to that employed to tag the *azgA* (Cecchetto *et al*., 2004) and *rrmA* (Olszewska *et al*., 2007). Starting from strain CS2778 (Li Destri Nicosia *et al*., 2001), which contains an *impala yA* ^+^ tagged element interrupting the *niaD* promoter, we obtained strain 2431A, *yA*Δ::*Ncpyr*4, *pabaA*1, *niaD^p^::impala::yA^+^ pacC* ^−/+^20205 by crossing. The *impala* excision frequency in strain 2431A was determined to be of the order of 10^−5^, similar to that reported for strain CS2778 (Li Destri Nicosia *et al*., 2001). Strain 2431A is unable to grow on nitrate by virtue of the *impala* element inserted in the *niaD* promoter and it is unable to grow at pH 7.5 due to the presence of the *pacC* ^−/+^20205 mutations. Thus, a strain able to grow on nitrate at pH 7.5 should result from simultaneous excision of the *impala* element and its insertion in *pacX*, which would suppress the phenotype of *pacC* ^−/+^20205. Conidiospores of strain 2431A were obtained on minimal media with NaNO_2_ as sole nitrogen source and plated by top layering on the appropriate selective media (NaNO_3_ as sole nitrogen source, pH 7.5, see above). One putative strain (BG2) carrying a transposon insertion in *pacX* was selected as able to grow in this medium. Based on the excision frequency of strain 2431A and the number of viable conidiospores plated, we calculated that ∼ 600 000 *niaD^+^* colonies were generated in the experiment that yielded the putative *pacX* insertion.

### Determining DNA sequence flanking the *impala* insertion site

Genomic DNA of strain BG2 was isolated and used as template to obtain DNA sequence flanking the *impala* insertion site by PCR using a single primer yA1 and cycling conditions as described by Karlyshev *et al*. (2000). The major fragment of ∼ 0.9 kbp was gel purified and sequenced using oligo yA2. The fragment was re‐amplified using primer yA1, digested with *Bam*HI (to obtain fragments having a single yA1 sequence), and the gel‐purified major fragment of ∼ 650 bp was sequenced using primer yA1. These sequencing reactions combined gave 708 bp of sequence flanking the insertion site. Primer sequences are given in Table S2.

### Determining the *pac* *X* genomic and cDNA sequences and *pac* *X* mutant sequence changes

BLAST search of the (then unfinished) *A. fumigatus* genome database (Nierman *et al*., 2005) suggested a possible *A. fumigatus* homologous gene having a C‐terminal zinc binuclear cluster. A larger *A. nidulans pacX* fragment was PCR amplified using the *pacX*‐specific primer XF2 and a degenerate primer ZNF2 based on the sequence of the zinc binuclear cluster of the putative *A. fumigatus* homologue. In the first three cycles, an annealing temperature of 45°C was employed, followed by 30 cycles at 55°C; 20 pmoles of XF2 and 200 pmoles of ZNF2 were used per reaction. The resulting ∼ 1.6 kbp fragment was gel purified and sequenced. A DNA fragment made by PCR using primers XF5 and XR5 was used to isolate *pacX* clones from a λ gt10 cDNA library (Osmani *et al*., 1988). Two cDNA clones contained the entire *pacX* coding sequence and sequencing confirmed the presence of one intron. Nucleotide sequence of the 5′ region of the *pacX* gene (upstream of the *impala* insertion and cDNA sequence) was obtained following an ‘inverse’ PCR strategy. Essentially, DNA of BAC clone 28C10 obtained from an *A. nidulans* BAC library prepared by Ralph Dean and obtained from https://www.genome.clemson.edu/online_orders?&page=productGroup&service=bacrc&productGroup=96 was digested with various restriction enzymes, purified and treated with T4 DNA ligase to circularise fragments. The DNA was PCR amplified using two (‘outward‐facing’) primers expected to give no product on linear DNA, XR6 and XF2, which resulted in∼ 1.8 and ∼ 2.8 kbp DNAs when BAC 28C10 DNA had been digested with *SacII* and *XhoI* respectively. The PCR fragments were sequenced using XR1 and additional primers.

Genomic DNA of *pacX* mutants was PCR amplified using *pacX*‐specific primers (e.g., XF2 and XR8), and the fragments were sequenced using additional gene specific primers. Mutations were, in most cases, confirmed on the opposite strand of a different PCR fragment.

Primer sequences are given in Table S1.

### *In silico* analyses

PacX orthologues were searched in the JGI (http://genome.jgi‐psf.org/programs/fungi/index.jsf) and NCBI databases (http://blast.ncbi.nlm.nih.gov/Blast.cgi). *A. nidulans Fot1*‐like elements were searched in http://www.aspgd.org/ (Cerqueira *et al*., 2014). Alignments were carried out with MAFFT version 7 5, http://mafft.cbrc.jp/alignment/server/ (Katoh and Standley, 2013), refinement with BMGE, http://mobyle.pasteur.fr/cgi‐bin/portal.py#forms::BMGE (Criscuolo and Gribaldo, 2010), Maximum Likelihood Phylogeny with PhyML, http://phylogeny.lirmm.fr/phylo_cgi/alacarte.cgi, (Dereeper *et al*., 2008; Guindon *et al*., 2010) calculating also approximate likelihood ratio tests (Anisimova and Gascuel, 2006). Tree drawing was done with Figtree (http://tree.bio.ed.ac.uk/software/figtree/). Coiled‐coil prediction was carried out with http://toolkit.tuebingen.mpg.de/pcoils (Parry, 1982; Lupas *et al*., 1991; Lupas, 1996). Conservation and putative functionality of individual PacX residues was calculated with ConSurf, http://consurf.tau.ac.il/ (Ashkenazy *et al*., 2010; Celniker *et al*., 2013). Nuclear localisation signals were searched with ngLOC http://genome.unmc.edu/ngLOC/index.html (King and Guda, 2007) PSORT II, http://psort.hgc.jp/form2.html (Nakao and Nakai, 2002), cNLS Mapper, http://nls‐mapper.iab.keio.ac.jp/cgi‐bin/NLS_Mapper_form.cgi (Kosugi *et al*., 2009) NetNes, http://www.cbs.dtu.dk/services/NetNES/ (La Cour *et al*., 2004).

### Microscopy and imaging techniques

For PacX localisation studies, the PacX::GFP HhoA::mCherry strain ALO2 was cultured at 28°C in LabTek chambers (Rochester NY) containing WMM [watch minimal medium (Peñalva, 2005)], adjusted to acid or alkaline pH as described (Galindo *et al*., 2007). Epifluorescence images were acquired with a Leica DMI6000 inverted optics microscope coupled to a Hamamatsu ORCA ERII camera, using Metamorph software (Molecular Devices) and SemrockBrightlinefilter sets for red and green fluorescence emission, essentially as described (Pantazopoulou and Peñalva, 2009; 2011). Z‐stacks of images were processed using the Metamorph ‘unsharp’ filter and, when needed, used to construct maximal intensity projections. Images were exported to Corel as TIFF maps.

## Supporting information

Supporting information
