# Supplementary material for: Refining the pH response in A spergillus nidulans: a modulatory triad involving PacX, a novel zinc binuclear cluster protein
Source: Mol Microbiol. 2015 Oct 16;98(6):1051–72. doi: 10.1111/mmi.13173 (PMC4832277; doi:10.1111/mmi.13173)
Supplement: Supplementary file 1 — Supporting information [file MMI-98-1051-s001.pdf]

Supplementary Information

Refining the pH response in *Aspergillus nidulans*: a modulatory triad involving PacX, a novel zinc binuclear cluster protein

Henk-Jan Bussink[1], Elaine M. Bignell[1,2], Tatiana Múnera-Huertas[1], Daniel Lucena-Agell[3], Claudio Scazzocchio[1,4], Eduardo A. Espeso[3], Margherita Bertuzzi[2], Joanna Rudnicka[1], Susana Negrete-Urtasun[1], Maria M. Peñas-Parilla[1], Lynne Rainbow[1], Miguel Á. Peñalva[3], Herbert N. Arst Jr.[1] and Joan Tilburn[1*]

[1] *Section of Microbiology, Imperial College London, Flowers Building, Armstrong Road, London SW7 2AZ, UK.*

[2] *Manchester Fungal Infection Group, Institute for Inflammation and Repair, University of Manchester, 46 Grafton Street, Manchester M13 9NT, UK.*

[3] *Department of Cellular and Molecular Biology, Centro de Investigaciones Biológicas CSIC, Ramiro de Maeztu 9, Madrid 28040, Spain.*

[4] *Institute for Integrative Biology of the Cell (I2BC), CEA, CNRS, Université Paris-Sud, Orsay, France.*

*Corresponding author

Joan Tilburn

Email j.tilburn@imperial.ac.uk

Telephone +1 240 271 4557

Running title: PacX and pH regulation refinement in *A. nidulans*

Key words: pH regulation, transcription, PacX, *Aspergillus nidulans*, zinc binuclear cluster, *palF* regulation.

Contents

Table S1. List of primers used in this work.

Fig. S1 (A). Epifluorescence microscopy of preferentially nuclear localized GFP-tagged PacC^{27} reveals that PacC^{27} levels are greatly reduced when co-expressed with PacC^{72} in a diploid strain.
(B). Reversal of epistasis relationships by over-expression of *alcA*p::PacC5-678 in a *pacC*^{c}14 *palA*1 strain.

Fig. S2. Electrophoretic Mobility Shift Assays (EMSAs) showing the effects of *pac*X1 on complexes formed by PacC.

Fig. S3. Coiled coil predictions for PacX and the consensus derived from an alignment of PacX homologues.

Fig. S4. ConSurf alignment of PacX homologues.

Fig. S5. Partial dominance of *pacX*1 to the wild type allele in a homozygous *palA*1 diploid.

Fig. S6. Phylogenic Tree of PacX homologues.

Supplementary Data

Fig. S7. Protein alignment: AN0826 ORF compared with known active fungal transposons.

Supplementary References

Table S1. List of primers used in this work

Primer name	primer sequence (5’ to 3’)
850U	GTCGCTCCTGTAGCTGTG
1217FF	GTTCGTGCTCTTTCGGCCA
AN0826F	CATGGTTTGCAGCCGCCA
AN0826R	GAAACTCAGATGCGGCCT
BIGFF	GCCGTTAAAACAGGCAGG
p1673-EcoRI-Fw	CGAGCTGTACAAGGAATTCCCCATGGCCGAAG
p1673-EcoRI-Rv	CTTCGGCCATGGGGAATTCCTTGTACAGCTCG
TILREV	CATTTCTCGTCCGCTCAT
XF2	CCGGGGGATTCAAGAAC
XF5	CAAAACAGAGTCGCGTGGAGAT
XR1	AAGGAGCAGAAAAGAGTCATGG
XR5	GTTCACCTCGCTGCAGTTC
XR6	CCAATGCGGGTTTAGATTCGG
XR8	GCGGAGTAGGATGTTGATTG
yA1	GGGAACCTGTCAACGCAAATC
yA2	GCCATACCCAGATCCCTTGAC
ZNF2	GGGGTACCKRCANCKRTCRCANGG

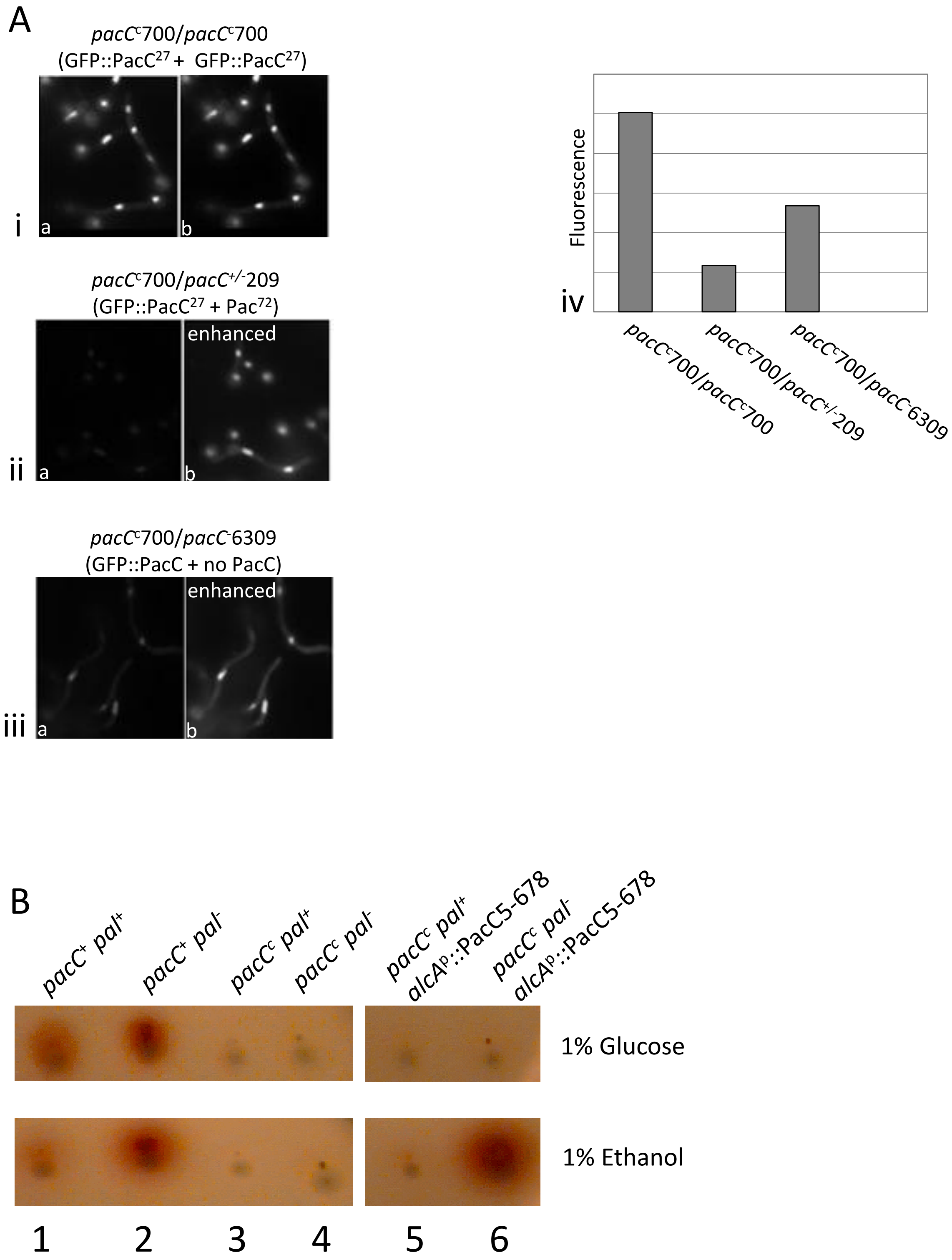
A
pacCc700/pacCc700
(GFP::PacC27 + GFP::PacC27)
i a b
pacCc700/pacC+/-209
(GFP::PacC27 + Pac72)
enhanced
ii a b
pacCc700/pacC-6309
(GFP::PacC + no PacC)
enhanced
iii a b
Fluorescence
iv
pacCc700/pacCc700
pacCc700/pacC+/-209
pacCc700/pacC-6309
B
pacC+ pal+
pacC+ pal-
pacCc pal+
pacCc pal-
pacCc pal+ alcAp::PacC5-678
pacCc pal- alcAp::PacC5-678
1% Glucose
1% Ethanol
1
2
3
4
5
6

Figure S 1

Fig. S1 (A). Epifluorescence microscopy of preferentially nuclear localized GFP-tagged PacC^{27} reveals that PacC^{27} levels are greatly reduced when co-expressed with PacC^{72} in a diploid strain. The *pacC*^{c}700 GFP tagged *pacC* constitutive allele (GFP::PacC5-250) corresponding to PacC^{27} is integrated at the *pacC* resident locus and expressed from the *pacC* promoter. The *pacC*$^{-}$6309 null mutation [specifying PacC(1-4)] reduced the fluorescence intensity of GFP::PacC5-250 to ~50% (ia or b, iiia and iv), which would be the expected dilution effect. The *pacC*$^{+/-}$209 processing recalcitrant mutation (specifying PacC^{72}) had a greater effect resulting in a reduction of fluorescence intensity to less than 25% of that found in the diploid *pacC*^{c}700 strain (Ia or b, iia and iv) or a haploid *pacC*^{c}700 strain (results not shown). These results are consistent with our proposal that PacC^{72} is a repressor of *pacC* expression. Enhanced images for diploids *pacC*^{c}700/*pacC*$^{+/-}$209 and *pacC*^{c}700/*pacC*$^{-}$6309 (iib and iiib, respectively) were obtained by modifying brightness and contrast to improve visualisation of the GFP::PacC5-250 protein to a level comparable to that in the *pacC*^{c}700 homozygous diploid (unenhanced, ia and b). Fluorescence images of haploid and diploid *pacC*^{c}700 cells (Fig. S1A) were taken using a DMI6000b microscope equipped with a 63x objective 1.4 N.A. and an Orca-ERII camera. Strains were cultivated in WMM [watch minimal medium (Peñalva, 2005)], containing the required supplements and glucose 1% and 5 mM ammonium tartrate as carbon and nitrogen sources, respectively. Cells were grown at 25^{o}C for 18 hours before epifluorescence analyses. To compare nuclear fluorescence intensities, all images were obtained in the same conditions with an exposure time of 2 seconds. Nuclear fluorescence intensities were estimated using Metamorph 6.3r software by measuring selected areas of 80 pixels into 10 nuclei per strain. The mean of accumulative pixel intensities for areas of 80 pixels are shown in the chart (n=10 nuclei). The diploid strains are *pacC*^{c}700/*pacC*^{c}700, *pabaA*1 *yA*2 *glrA*1 *pacC*^{c}700 / *lysA*2 *pacC*^{c}700 (J2000); *pacC*^{c}700/*pacC*$^{+/-}$209, *lysA*2 *pacC*^{c}700 / *biA*1 *sB*3 *pacC*$^{+/-}$209 (J1977) and *pacC*^{c}700/*pacC*$^{-}$6309, *pabaA*1 *yA*2 *glrA*1 *pacC*^{c}700 / *pacC*$^{-}$6309 (*pacC*^{c}63) *pantoB*100 (J1999).

(B). Reversal of epistasis relationships by over-expression of *alcA*p::PacC5-678 in a *pacC*^{c}14 *palA*1 strain. Acid phosphatase staining after growth on minus phosphate medium pH 6.5 is shown. The *pacC*^{c}14 and *palA*1 alleles were used. The *alcA*^{p}::PacC5-678 cassette was integrated at the *argB* locus. In haploid strains *pacC*^{c} mutations are epistatic to *pal*$^{-}$ mutations (Caddick *et al*., 1986; Denison *et al*., 1995; Orejas *et al*., 1995), as shown by reduced acid phosphatase staining in positions 3 and 4. However, in a merodiploid strain containing *alcA*^{p}::PacC5-678 integrated in *argB* in addition to *pacC*^{c}14, *pal*A1 which is hypostatic to *pacC*^{c}14 on glucose medium becomes epistatic to *pacC*^{c}14 when PacC5-678 is over expressed under *alcA*^{p} inducing conditions (1% ethanol) lane 5. The strains containing the transgene were obtained by transformation (Tilburn *et al*., 1983) of a strain *pabaA*1 *yA*2 *argB*2 *pacC*^{c}14 or *yA*2 *argB*2 *palA*1 *pacC*Δ::*Ncpyr4 pantoB*100 with plasmid p[*alcA*^{p}::PacC5-678] (Mingot *et al*., 1999) and crossing. Strains in lanes (1) wildtype *biA*1 (J734 or 2167A), (2) *biA*1 *palA*1 (BXS), (3) *pabaA*1 *pacC*^{c}14 (J542), (4) *biA*1 *pabaA*1 *argB*::*alcA*^{p}::PacC5-678) *pacC*^{c}14 (J601), (5) *yA*2 *pabaA*1 *palA*1 *argB*::*alcA*^{p}::PacC5-678 *pacC*^{c}14 *pantoB*100 (J776).

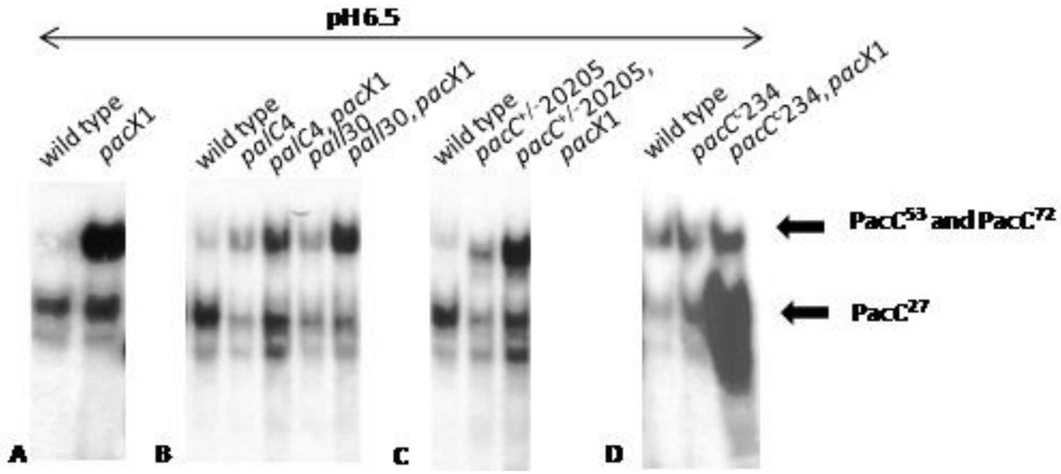

Fig. S2

Electrophoretic Mobility Shift Assays (EMSAs) showing the effects of *pac*X1 on complexes formed by PacC in *pac*C$^+$ (i), *pal*$^-$ (ii), *pacC*$^{+/-}$20205 (iii), and *pacC*^{c}234 backgrounds. PacC was detected using a ^{32}P-labelled oligonucleotide containing *ipn*A2 (Tilburn *et al*., 1995). The positions of the PacC forms are indicated. Each binding reaction contained 5 μg of crude protein. Strains were grown from conidial inocula of 1 - 2 x 10^6 ml^{-1} in 200 ml of appropriately supplemented *Aspergillus* complete media (Cove, 1966), containing 3% ($^w/_v$) sucrose, 20 mM MES pH 6.5, at 37°C for 16 hours with shaking at 120 rev min^{-1}. Mycelia were harvested on sterile Miracloth™ (Calbiochem) and samples of mycelia were cut into small strips of approximately 300 mg and frozen in liquid nitrogen. Cell lysates were prepared essentially as described by Peñas *et al.* (2007) except that the samples were centrifuged immediately after cell disruption. Double stranded oligonucleotide probes were prepared and binding reactions and electrophoresis were carried out following Perez-Esteban *et al.* (1993) and Espeso and Peñalva (1994).

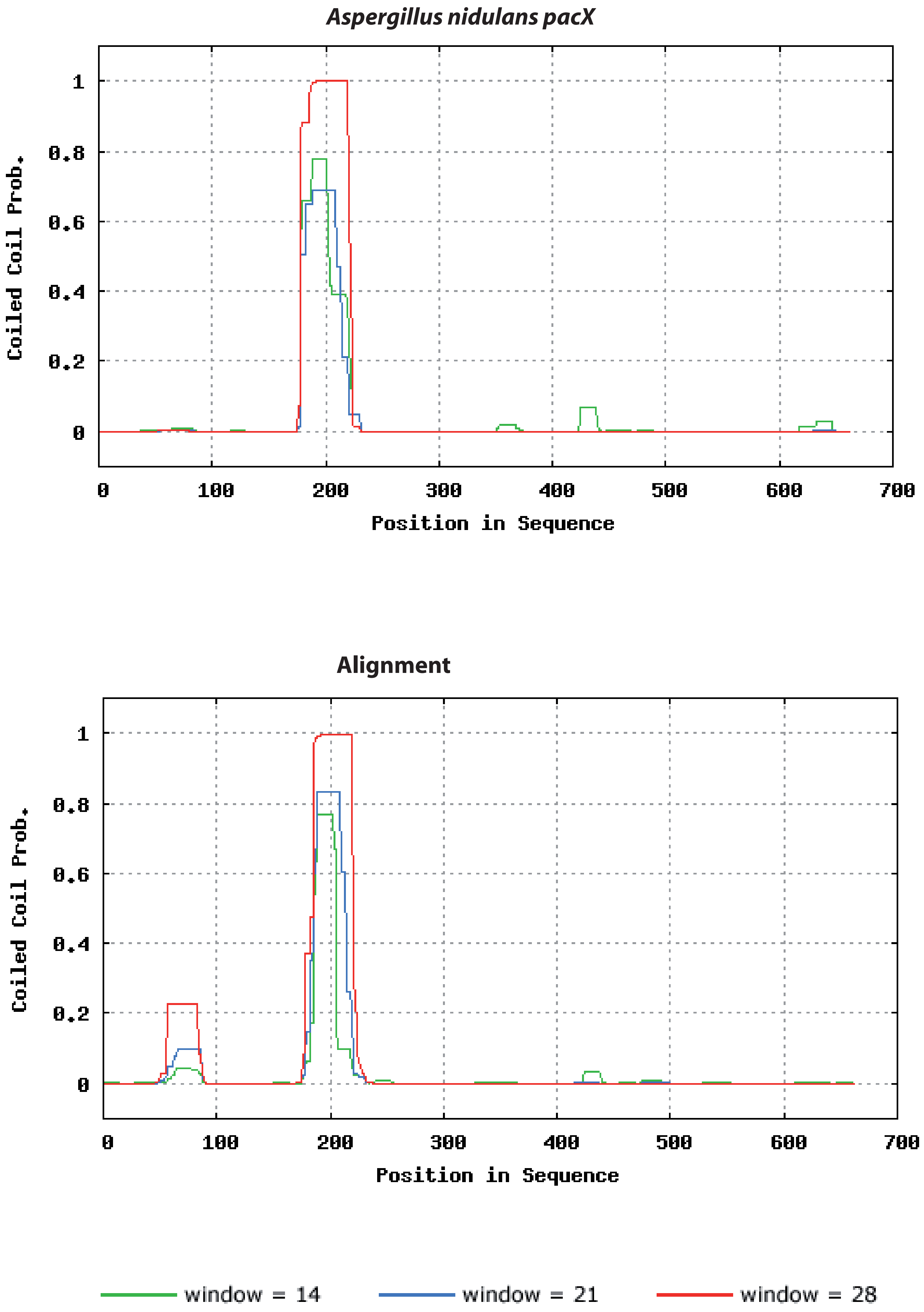
Aspergillus nidulans pacX
Coiled Coil Prob.
1
0.8
0.6
0.4
0.2
0
0
100
200
300
400
500
600
700
Position in Sequence
Alignment
Coiled Coil Prob.
1
0.8
0.6
0.4
0.2
0
0
100
200
300
400
500
600
700
Position in Sequence
window = 14
window = 21
window = 28

Fig. S3. Coiled-coil predictions for PacX and the consensus derived from a 177-membered alignment of PacX homologues were obtained at http://toolkit.tuebingen.mpg.de/pcoils. The same alignment used to generate the phylogeny shown in Fig.S6 was used to obtain the coiled-coil prediction.

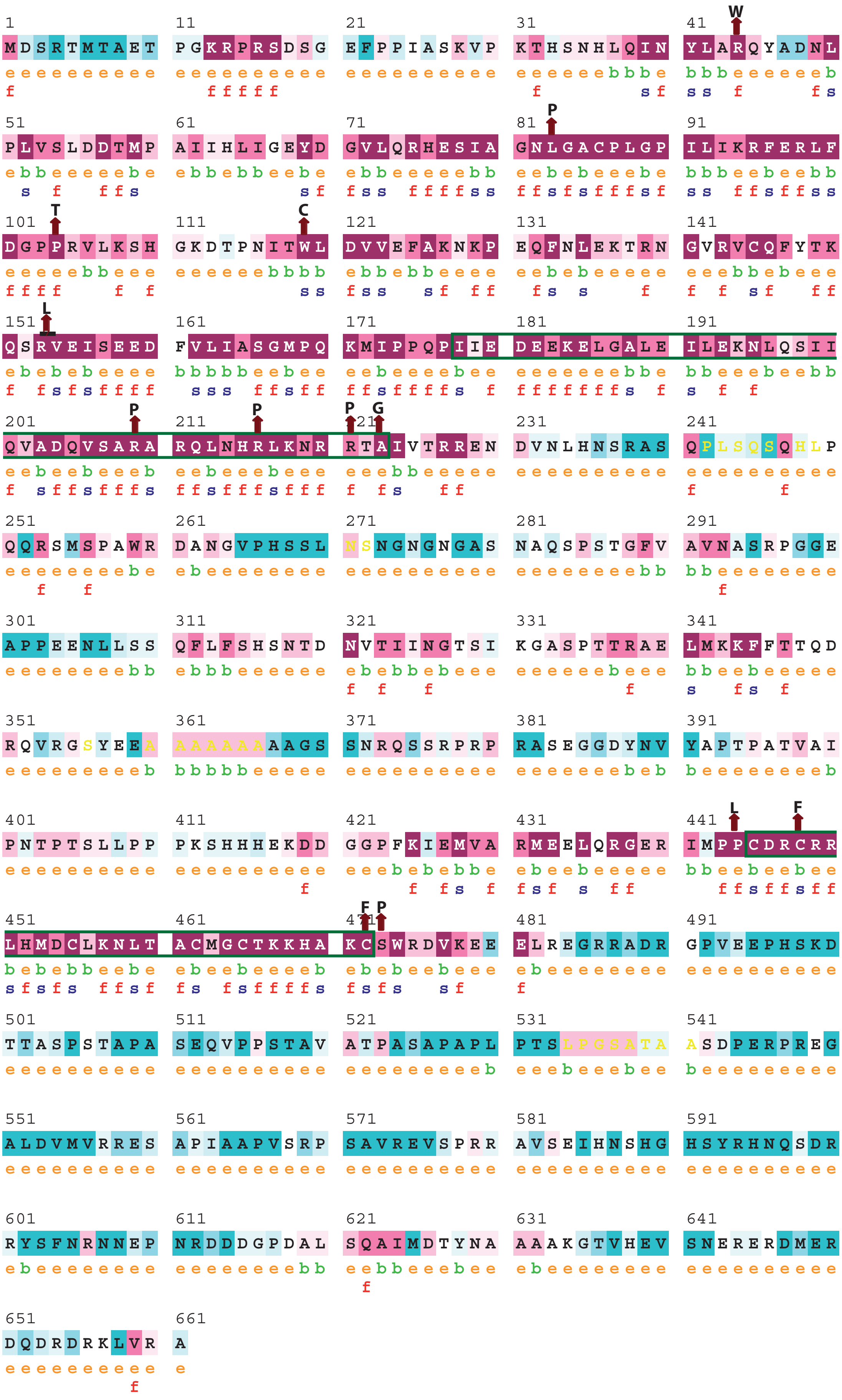

The conservation scale:

1 2 3 4 5 6 7 8 9

Variable Average Conserved

e - An exposed residue according to the neural-network algorithm.

b - A buried residue according to the neural-network algorithm.

f - A predicted functional residue (highly conserved and exposed).

s - A predicted structural residue (highly conserved and buried).

X - Insufficient data - the calculation for this site was performed on less than 10% of the sequences.

Fig. S4. ConSurf (http://consurf.tau.ac.il) results for PacX based on the 177-membered alignment used to generate the phylogeny in Fig. S6. Residues where point mutations map are indicated with dark arrows and residue changes are shown. The coiled-coil region and zinc binuclear cluster are outlined in green.

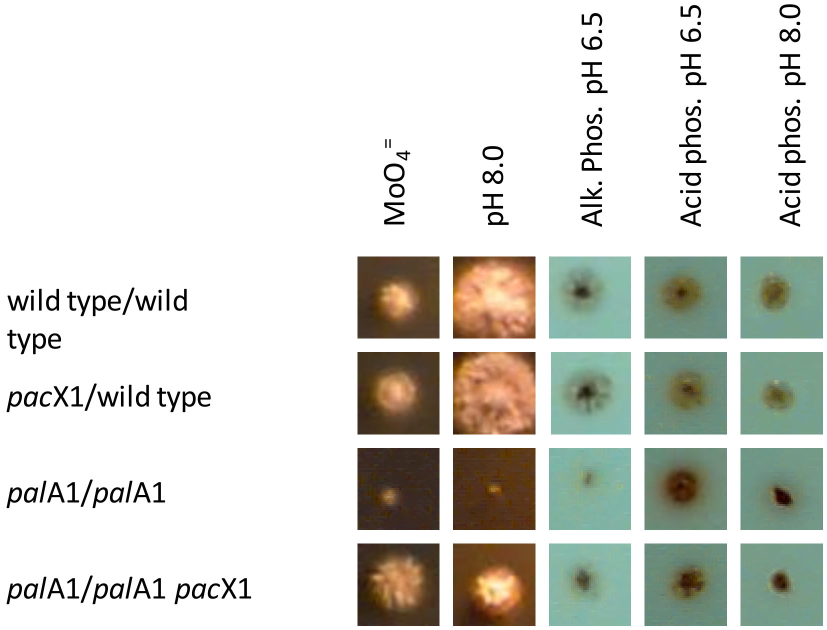

Fig. S5. Partial dominance of *pacX*1 to the wild type allele in a homozygous *palA*1 diploid. Diploids of relevant partial genotype are indicated. Growth on 25 mM molybdate and pH 8.0 medium are shown. Alkaline and acid phosphatase staining was carried out after growth on minus phosphate medium, buffered at the pH values shown, for ~24h at 37°C.

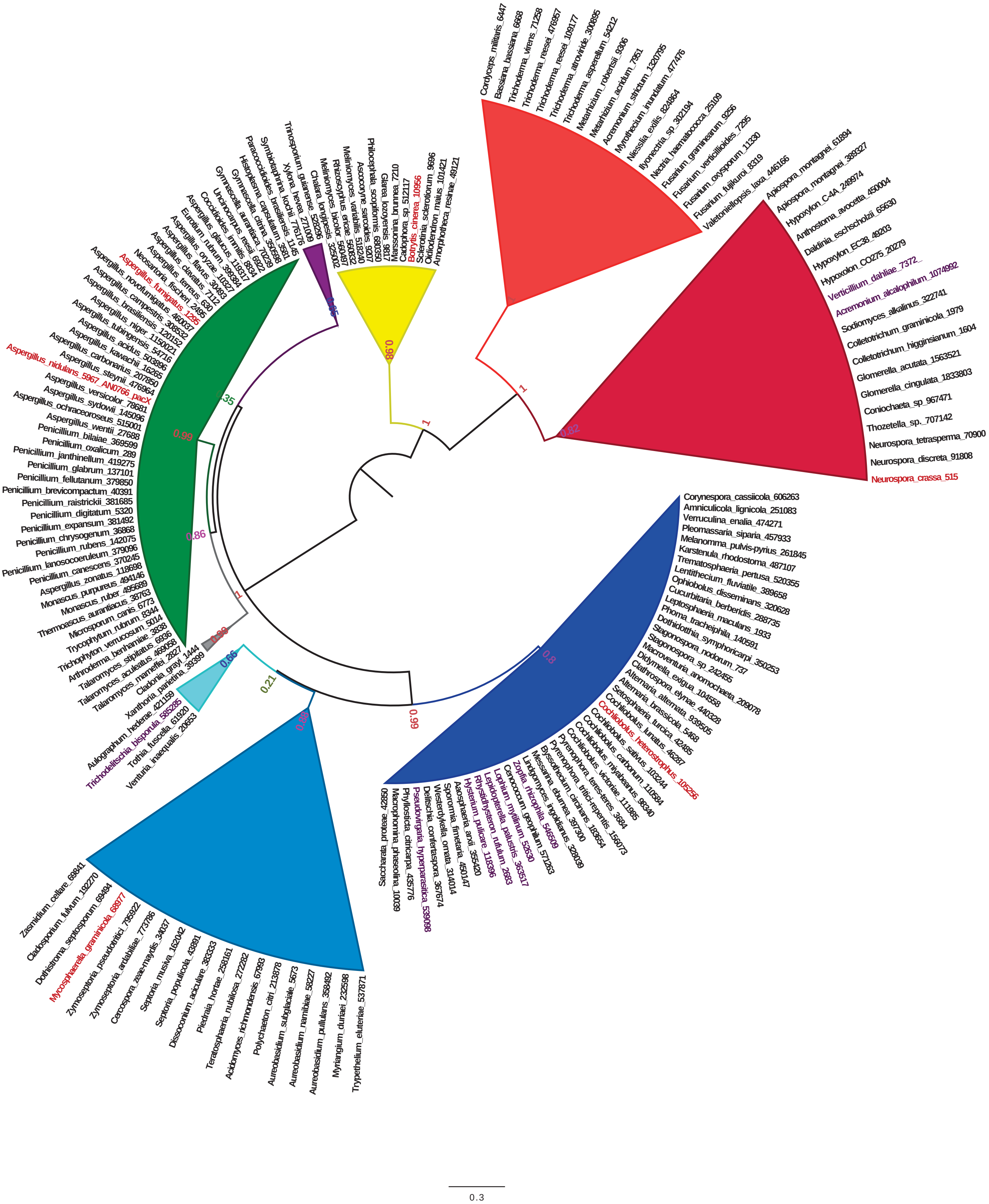
Cordyceps_militaris_6447
Beauveria_bassiana_6668
Trichoderma_virens_71258
Trichoderma_reesei_476957
Trichoderma_reesei_109177
Trichoderma_atroviride_300895
Trichoderma_asperellum_54212
Metarhizium_robertsii_9306
Metarhizium_acridum_7951
Acremonium_strictum_1320795
Myrothecium_inundatum_477476
Niesslia_exilis_824864
Ilyonectria_sp_302194
Nectria_haematococca_25109
Fusarium_graminearum_9256
Fusarium_verticillioides_7295
Fusarium_oxysporum_11330
Fusarium_fujikuroi_8319
Valetoniellopsis_laxa_446166
Apiospora_montagnei_61894
Apiospora_montagnei_389327
Hypoxylon_C-4A_249974
Anthostoma_avocetta_450004
Daldinia_eschscholzii_65630
Hypoxylon_EC38_48203
Hypoxylon_CO275_20279
Verticillium_dahliae_7372_
Acremonium_alcalophilum_1074992
Sodiomyces_alkalinus_322741
Colletotrichum_graminicola_1979
Colletotrichum_higginsianum_1604
Glomerella_acutata_1563521
Glomerella_cingulata_1833803
Coniochaeta_sp_967471
Thozetella_sp._707142
Neurospora_tetrasperma_70900
Neurospora_discreta_91808
Neurospora_crassa_515
Corynespora_cassiicola_606263
Amniculicola_lignicola_251083
Verruculina_enalia_474271
Pleomassaria_siparia_457933
Melanomma_pulvis-pyrius_261845
Karstenula_rhodostoma_487107
Trematosphaeria_pertusa_520355
Lentithecium_fluviatile_389658
Ophiobolus_disseminans_320628
Cucurbitaria_berberidis_288735
Leptosphaeria_maculans_1933
Phoma_tracheiphila_140591
Dothidotthia_symphoricarpi_350253
Stagonospora_nodorum_737
Stagonospora_sp_242455
Macroventuria_anomochaeta_209078
Didymella_exigua_104558
Clathrospora_elynae_440328
Alternaria_alternata_939505
Alternaria_brassicicola_5468
Setosphaeria_turcica_42485
Cochliobolus_lunatus_46287
Cochliobolus_heterostrophus_105256
Cochliobolus_sativus_103244
Cochliobolus_carbonum_110584
Cochliobolus_miyabeanus_98240
Cochliobolus_victoriae_111985
Pyrenophora_teres-teres_3684
Pyrenophora_tritici-repentis_156073
Byssothecium_circinans_183654
Massarina_eburnea_397300
Lindgomyces_ingoldianus_326939
Zopfia_rhizophila_546509
Cenococcum_geophilum_571263
Lophium_mytilinum_52630
Lepidopterella_palustris_363517
Rhytidhysteron_rufulum_2683
Hysterium_pulicare_118396
Aaosphaeria_arxii_355420
Sporormia_fimetaria_450147
Westerdykella_ornata_314014
Delitschia_confertaspora_367674
Pseudovirgaria_hyperparasitica_539098
Phyllosticta_citricarpa_435776
Macrophomina_phaseolina_10039
Saccharata_proteae_42850
Zasmidium_cellare_69841
Cladosporium_fulvum_192270
Dothistroma_septosporum_69494
Mycosphaerella_graminicola_68877
Zymoseptoria_pseudotritici_795922
Zymoseptoria_ardabiliae_773786
Cercospora_zeae-maydis_34037
Septoria_musiva_162042
Septoria_populicola_43891
Dissoconium_aciculare_383333
Piedraia_hortae_258161
Teratosphaeria_nubilosa_272282
Acidomyces_richmondensis_67993
Polychaeton_citri_213878
Aureobasidium_subglaciale_5673
Aureobasidium_namibiae_58227
Aureobasidium_pullulans_358482
Myriangium_duriaei_232598
Trypethelium_eluteriae_537871
Venturia_inaequalis_20653
Tothia_fuscella_61920
Trichodelitschia_bisporula_585285
Aulographum_hederae_421159
Xanthoria_parietina_39399
Cladonia_grayi_1444
Talaromyces_marneffei_2827
Talaromyces_aculeatus_469058
Talaromyces_stipitatus_6936
Arthroderma_benhamiae_3838
Trichophyton_verrucosum_5014
Trycophyton_rubrum_8344
Microsporum_canis_6773
Thermoascus_aurantiacus_38763
Monascus_ruber_495689
Monascus_purpureus_494146
Aspergillus_zonatus_118698
Penicillium_canescens_370245
Penicillium_lanosocoeruleum_379096
Penicillium_rubens_142075
Penicillium_chrysogenum_36868
Penicillium_expansum_381492
Penicillium_digitatum_5320
Penicillium_raistrickii_381685
Penicillium_brevicompactum_40391
Penicillium_fellutanum_379850
Penicillium_glabrum_137101
Penicillium_janthinellum_419275
Penicillium_oxalicum_289
Penicillium_bilaiae_369599
Aspergillus_wentii_27688
Aspergillus_ochraceoroseus_515001
Aspergillus_sydowii_145096
Aspergillus_versicolor_78681
Aspergillus_nidulans_5967_AN0766_pacX
Aspergillus_steynii_476964
Aspergillus_carbonarius_207850
Aspergillus_kawachii_16265
Aspergillus_acidus_503896
Aspergillus_tubingensis_54716
Aspergillus_niger_1150021
Aspergillus_brasiliensis_120152
Aspergillus_campestris_308532
Aspergillus_novofumigatus_460037
Aspergillus_fumigatus_1295
Neosartorya_fischeri_2495
Aspergillus_clavatus_630
Aspergillus_terreus_7112
Aspergillus_flavus_30493
Aspergillus_oryzae_10327
Eurotium_rubrum_399384
Aspergillus_glaucus_119317
Coccidioides_immitis_8834
Uncinocarpus_reesii_6822
Gymnascella_aurantiaca_70239
Gymnascella_citrina_359598
Histoplasma_capsulatum_3581
Paracoccidioides_brasiliensis_1145
Symbiotaphrina_kochii_776176
Xylona_heveae_271009
Trinosporium_guianense_528286
Chalara_longipes_325002
Meliniomyces_bicolor_560497
Rhizoscyphus_ericae_518240
Meliniomyces_variabilis_560832
Ascocoryne_sarcoides_9207
Phialocephala_scopiformis_680350
Glarea_lozoyensis_9617
Marssonina_brunnea_7210
Cadophora_sp_512117
Botrytis_cinerea_10956
Sclerotinia_sclerotiorum_9696
Oidiodendron_maius_101421
Amorphotheca_resinae_49121
1
0.82
1
0.98
0.95
0.35
0.99
0.86
1
0.99
0.66
0.21
0.98
0.99
0.8
0.3

Fig. S6. Maximum likelihood phylogeny of 177 putative PacX orthologues. The tree is shown in a cartoon, circular form. The name of each species is indicated, followed by the protein accession number in the JGI data base. The classes and orders within the Pezizomycotina are colour coded as follows: Blue, Dothideomycetes, Pleosporales; LIght blue, Dothideomycetes, Capnodiales; Very light blue; Dothideomycetes, *Incerta sedis*; Grey, Leotiomycetes; Green, Eurotiomycetes; Purple, Xylonomycetes;Yellow, Leotiomycetes; Pink Sordariomycetes, Hypocreales, Red, Sordariomycetes, other orders. Names in purple indicate species that map outside their taxonomic order (but within its class); e.g. *Acremonium alcalophilum* does not map together with other Hypocreales, such as *Acremonium strictum, Trichodelitschia bisporula* does not map with other Pleosporales. Species in red are those used in Fig. 6. Alignment carried out with MAFFT E-INS-i, (recommended for sequences with multiple conserved domains and long gaps) with a Blosum 30 matrix, alignment refinement carried out with BMGE with a Blosum 30 similarity matrix. Tree generated with PhyML and re-drawn with Figtree. Support values in the nodes are aLRT (approximate likelihood ratio tests, see **Experimental Procedures**).

Supplementary Data

Inactivation of the *pacX* gene by an endogenous *Fot1*-like transposon

PCR amplification of *pacX*18, using primers XF2 and XR8, showed this mutation to result from an insertion of ~1.7 kb. Complete sequencing showed the insert to be 1860 bp (not including the characteristic TA duplication) with an open reading frame identical to that of AN0826, which is annotated in the http://www.aspergillusgenome.org data base as "Uncharacterised transposable element gene". The open reading frame of 1622 nt is bounded by 44 nt inverted repeats, the 5' terminating 50 nt upstream of the ATG, the 3' initiating 57 nt downstream from a double ochre chain termination signal. These features are identical to those flanking AN0826. The element is integrated in an orientation opposite to that of the open reading frame of *pacX* between the T and A of the acceptor site of the intron of *pacX*, and it generated a typical TA duplication. The open reading frame shows an obvious similarity with that of transposons of the *Fot1*/*pogo* family (Supplementary Fig. S7) shows the similarity of the peptidic sequences.

There are sixteen sequences in the *A. nidulans* genome which show high similarity with AN0826, with complete or incomplete open reading frames, all bounded by identical (in one case incomplete, AN7516) inverted repeats. These are namely, in order of similarity: AN4391, AN6559, AN3478, AN11091, AN4877, AN5334, AN0669, AN7516, AN2732, AN0975, AN8788, AN11044, AN3047, AN2291, AN5863, AN9046 (Cerqueira *et al*., 2014). Even the most similar sequences show sufficient nucleotide differences with AN0826 to make the identification of the inserted element certain. Southern blots had already revealed a minimum of eleven copies in the standard Glasgow laboratory strain (Li Destri Nicosia *et al.*, 2001) The most conserved open reading frames (AN0826 to AN3478, also AN11044) are not predicted to be interrupted by introns. In other ORFS introns are predicted. While we have not checked manually all putative ORFs, these introns are probably spurious, and are derived by automatic annotation from the presence of chain termination codons inactivating some of the ORFs. The variability in the position of

the putative intron within the different ORFs supports this contention. The case of AN8788 is glaring, a 472 nt putative intron results in an overlap of the proposed ORF with convergently transcribed AN8789 and would place the putative C-terminal exon beyond the inverted repeat. In this case a -2 frame shift has resulted in a truncated protein at residue 595, automated intron recognition process "fabricating", in fact, two introns.

It is striking that among the hundreds of loss-of-function mutations obtained by classical genetics and now sequenced, *pacX*18 is the first and only one due to the insertion of an endogenous transposon. Attempts to select specifically such events were never successful in *A. nidulans*. This contrasts with the ready mobility of heterologous *Fot1*, *impala* and *Minos* transposons inserted in the genome of *A. nidulans* (Li Destri Nicosia *et al.*, 2001; Carr *et al.*, 2010; Evangelinos *et al*., 2015)

An explanation for the extreme rarity of endogenous transposition in *A. nidulans* may derive from the absence of active transposase in the cell. Caddick and co-workers have carried out global RNAseq of the *A. nidulans* transcriptome under 5 different conditions (Sibthorp *et al.*, 2013). These data are accessible on line (http://www.aspgd.org/, JBrowse). A search for each one of the paralogues indicated above showed virtually (and in some cases absolutely) no transcripts. Some transcripts are seen for AN9604, the most divergent of the homologues, which cannot encode an active transposase. In contrast with this data, a signal for a transcript of the correct length was detected in early experiments by conventional Northern blots (Li Destri Nicosia *et al.*, 2001) in culture conditions similar to one of those used by (Sibthorp *et al.*, 2013). The probe used was derived from the only *A. nidulans Fot*-like element known at the time, whose position within a sequenced cosmid, F2P08, (Kupfer *et al.*, 1997) identifies it unequivocally as AN0975. The high similarity between all *Fot1*-like paralogues implies that cumulative transcription of all Fot-1 like elements in the genome were detected in this experiment.

The different homologues are spread throughout the genome, are not methylated (Li Destri Nicosia *et al.*, 2001), and active transcription of neighbouring genes show that they are not located in silent regions of the genome. It would be interesting to know if the absence of transcripts derives from very infrequent transcription or from selective degradation of the cognate RNAs though the dicer/argonaute system (Hammond *et al.*, 2008a and b). The strain in which *pacX18* was isolated carries an autonomous copy of an *impala* element from *F. oxysporum,* belonging to the *Tc1*/*mariner* family. While the cognate transposase cannot mobilise a *Fot1*-like element, the possibility remains that its presence may affect the regulation of its expression. In addition, possible effects of the *pacC*$^{+/-}$20205 mutation and the stressful alkaline growth conditions under which the *pacX*18 mutation was selected may ,have contributed to the activation of the AN 0826 *Fot-1-like* element

Transposons of the *Fot1*/*Pogo* family operate through a mechanism of cut and paste, rather than the copy and paste characteristic of retroposons, and among the eukaryotic DNA transposons, of helitrons. We thus tested the presence of the resident copy of AN0826 in the strain where transposition to *pacX* had occurred. Fig S7 shows clearly that AN0826 is conserved at its locus in the strain carrying the *pacX*18 mutation, thus demonstrating a copy/paste mechanism, however, the precise insertion of the inverted repeats and the TA duplication implies that this event was catalysed by the specific transposase.

That copy and paste expansion of eukaryotic type II transposons must occur is indicated by the multiple copies of these elements in genomes where they are present (Daboussi and Capy, 2003; Dufresne *et al.*, 2011, specifically for *Fot1* elements), and in the specific case of the *Fot1*-like transposons of *A. nidulans* by their polymorphism in different strains of this species (Li Destri Nicosia *et al.*, 2001). A class II "cut and paste" DNA transposon can replicate by transposing from a position behind a replication fork to an un-replicated sequence or by double-stranded gap repair at the donor site by an intact sister chromatid (Rubin and Levy, 1997; Bessereau, 2006; Wicker *et al.*, 2007; Izsvak *et al.*, 2009; Skipper *et al.*,

2013 and refs therein). Both mechanisms imply a temporal coupling of replicative transposition and the S phase of the cell cycle. AN0826 and *pacX* are both on chromosome VIII at ~180 kb from each other, which is not incompatible with these genes being within the same replicon. This proximity may have facilitated a local transposition event [local hopping, (Tower *et al.*, 1993; Timakov *et al.*, 2002; Carlson and Largaespada, 2005; Muñoz-Lopez and García-Pérez, 2010)] and allowed us to detect this very rare occurrence.

```
AN0826    1 MPRVRVSSSQNCHEKEGRLLLAVQAIKKKEITSIREAARRFNVPESTLRTRLRGTTNRAESRA-NGHKLTEIEEEVLKQW
Tan1      1 MPPKASIPSKSQVEQEGRILLAIEAIQKGQITSIREAARVYDVARTTLQARLSGRVFAKNMTN-ARQKLSNNEEESLVKW
Fot1      1 MPVYSADDLEN----------AIADFKNG--VSLKTAAKKNGLPPSTLRGRLTGAQSRQVARQ-EQLRLTTDQEDDLERW
Aft1      1 MPKSSKI-------NESYLLEACEAAQAQKKPNISKIAREYGVPYATLRDRVKKHVHPRLANKPVNRALKGYQEEALIQW

AN0826   80 ILSLDLRGAAPTKAHVREMANILLAKRGSTPIQTVGQKWVYNYTQRHPE---LESRLSRQYDCQRAKQENPKVIQAWFNT
Tan1     80 ILSLDKRGASPRPLDIRDMANLIISKRGYSTVEQVGINWAYSFVKRHES---LRTRFARRLNYQRAKMEDPEVIKDWFKR
Fot1     68 ILRQEKLGHAPTHAQVRTIVRSVLARHGDH--APLGRKWTTRFVERHPA---LKTKLGRRTDWERVNAATPANIKRLFDV
Aft1     74 IVCMRDRNMPVTPKLLEEYANQALRRAGES--RQVSKMWAYRFEKRLPEHLNLGPAKQKIKESKRIQAEDAGLLTHWYNQ

AN0826  157 VRATIEQYGILPDDIYNFDETGFAMGLCAHQKVITKSE-SCGRRPVLQPGNR-EWVTAIESISASGWAL-PILIFKGKQ-
Tan1    157 VQEVIQEYGISSDDIYNFDETGFAMGMIATYKVVTSSQ-RAGRPSLVQPGNR-EWVTAIECIRSNGEVLPSTLIFKGKT-
Fot1    143 YETVD---WIPPERRYNADEGGIMEGQGVNGLVIGSSQESPNAVPVKTATVR-TWTSIIECISAVGVVLHPLVIFKAKT-
Aft1    152 LAGVVKK-DTPARLVYNFDECGFQPGEGKSRKVISS---KGSKVPDLAESERGENITAIECVAADGWQMDPWFIFKGNGI

AN0826  233 YNQAWF-TG--LPP--DWRFEISTNGWTTNEISLRWLQKQFIPSTEHRTRGRYQLLVLDGHGSHLTPEFDQICTDHNIIP
Tan1    234 HLKAWY-EGQSIPP--TWRFEVSDNGWTTDKIGLRWLQKHFIPLIRGKSVGKYSLLVLDGHGSHLTPEFDQSCAENEVIP
Fot1    218 IQEQWF-RREFLQKHLGWQVTFSKNGWTSNSIALEWLEKVFLPQTAPADPADARLLIVDGHGSHATEQFMAKCYLNNVYL
Aft1    228 FMESWFNESEALPP--DTTIATSPNGWISDELAVQWLQSFINATNERTKKGEKRILIFDGHGSHLTVEFLQLCEDNGVIP

AN0826  308 LCMPAHSSHLLQPLDIGCFAVLKRSYASLVDQKMRLG-ISHIDKLDFLAAYPQARISTFKLDTIRNSFRAAGLVPLNPEP
Tan1    311 ICMPAHSSHLLQPLDVGCFSVLKRTYGGMVQKQMQYG-RNHIDKLDFLEVYPKAHQCALSKSNIISGFRATGLVPLDPDQ
Fot1    297 LFLPAHCSHVLQPLDLGCFSSLKAAYRTLVGEHTALTDSTRVGKQRFLDFYARAREIGFRKVNIRSGWRAAGLWPVNINK
Aft1    306 FGFLPHTTHLCQPLDGKPFLSYKQHFRRMNNELSYWA-GEPVGKSEFLHMIGPVREKAFNQRIIREAFKDRGIWPVN-SK

AN0826  387 VLSKLSIQA--------------CTPTPPG----SRGSQASTFCPHTPANVDELLKQASLLRDFLKQRSKSPPSPSHNAL
Tan1    390 VLSRLHIRL--------------KTPPTPD-------SQSSGSVLQTPHNIKHLLKHQKSVERLLRKRQASPTSPTNSTL
Fot1    377 PLASRWVMVL-------------TKSALPP---------SETLDIATPKRGGDVVK-------LFSAKSSSPSSRL--SI
Aft1    384 IADDLAILLWEGIPDIYAPDLDKMTPSTPPSQPPSRPPSSSSIDISPPRTIQALKKNQAK----LSKHADLLTPKLQRNL

AN0826  449 NQLIKGCQIAMQKGILLEQENRALRAENAIQRRK-RARTHRW----IAHDNGLSVQEA----TE--LEEAHNASF-QAIP
Tan1    449 RQLLKGCELAITNSIILAKENAELRASHEKQLQK-RKRSRKQ----VIYTEGTTVEEAQRAIQE--VEEVQNDED-IEVE
Fot1    426 RKAAAALDKVAIELAMKDREIERLRAQLEAAQPK-KKRKIRQDPNECFISLAQILAEANREPDQRVIQSQKGDLDCIVVD
Aft1    460 ERIFEHNRIAAEHLAIANETIGRIRAAQAPLRRQYTKRQVKP----LSQSGILTLRDANRSIAS---------------

AN0826  517 GPCGPPAEGAQTPKARALPT-CSTCHRIGHRRNAC---PNK
Tan1    521 ----PQSQYTETP-SRAPPR-CSNCFNIGHRRTQCSKPPTN
Fot1    505 GKSSSESEEDPAPVRRSTRVRRATKMYIRQDLSSE---ESD
Aft1    520 -----RKAKDAAAQERRLQT---QWEKVHGKPPPL---AST
```

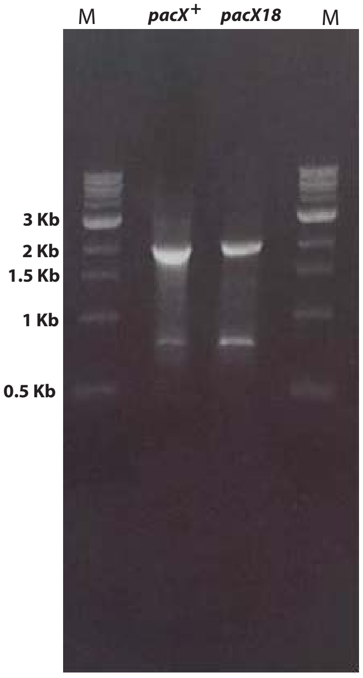


```
acgtggttggtaagcgagctgcgcatgtaagcgagctgcgcacc
||||||||||||||||||||||||||||||||||||||||||||
tgcaccaaccattcgctcgacgcgtacattcgctcgacgcgtgg
```

Fig. S7. Protein alignment: AN0826 ORF compared with known active fungal transposons of the *Fot1*/*Pogo* family, *Tan1* from *A. niger* (Nyyssönen *et al.*, 1996), *Aft1* from *A. fumigatus* (Hey *et al.*, 2008) and *Fot1* from *Fusarium oxysporum* (Daboussi *et al.*, 1992). Alignment carried out with MAFT, G-INS-i, visualised with Box-Shade (http://www.ch.embnet.org/software/BOX_form.html). Nucleotide alignment: inverted repeat (without the TA duplication) of AN0826 at its genomic locus and within the *pacX* gene in the *pacX*18 mutation. Inverted repeats detected with einverted: (http://emboss.bioinformatics.nl/cgibin/emboss/einverted). Right panel, PCR amplification with primers external to the AN0826 inverted repeats at its chromosomal location. M, molecular size markers. *pacX*$^{+}$ is the parent strain XC34 (*yA*Δ::*Ncpyr4 pabaA*1 *pacC*^{C}202 *pacC*$^{+/-}$20205 *pantoB*100 *niaD*Δ*::impala::yA*$^{+}$) for the *pacX*18 derivative (strain A869). The expected size of the amplified sequence would be 2118 bp in the *pacX*$^{+}$ strain and 456 bp if the *pacX*18 mutation resulted from a Cut and Paste mechanism. In the event amplified bands in both strains are of identical size, showing a Copy and Paste mechanism to be operating. Primers used for amplification, AN0826F and AN0826R.

Supplementary References

Bessereau, J.L. Transposons in *C. elegans*.(January 18, 2006) *WormBook*, ed. The *C. elegans* Research Community, WormBook, doi/iv.1895/wormbook.1.70.1, http://www.wormbook.org.

Caddick, M.X., Brownlee, A.G., and Arst,H.N., Jr. (1986) Regulation of gene expression by pH of the growth medium in *Aspergillus nidulans*. *Mol Gen Genet* **203**: 346-353.

Carlson, C.M., and Largaespada, D.A. (2005) Insertional mutagenesis in mice: new perspectives and tools. *Nat Rev Genet* **6**: 568-580.

Carr, P.D., Tuckwell, D. Hey, P.M. Simon, L., D'Enfert, C., Birch, M. *et al.* (2010) The transposon impala is activated by low temperatures: use of a controlled transposition system to identify genes critical for viability of *Aspergillus fumigatus*. *Eukaryot Cell* **9**: 438-448.

Cerqueira, G.C., Arnaud, M.B., Inglis, D.O., Skrzypek, M.S., Binkley, G., Simison, M. *et al.* (2014) The *Aspergillus* Genome Database: multispecies curation and incorporation of RNA-Seq data to improve structural gene annotations. *Nucleic Acids Research* **42 (Database issue)**: D705-D710.

Cove, D.J. (1966) The induction and repression of nitrate reductase in the fungus *Aspergillus nidulans*. *Biochim Biophys Acta* **113**: 51-56.

Daboussi, M.J., and Capy, P. (2003) Transposable elements in filamentous fungi. *Annu Rev Microbiol* **57**: 275-299.

Daboussi, M.J., Langin, T., and Brygoo, Y. (1992) Fot1, a new family of fungal transposable elements. *Mol Gen Genet* **232**: 12-16.

Denison, S.H., Orejas, M., and Arst, H.N., Jr. (1995) Signaling of ambient pH in *Aspergillus* involves a cysteine protease. *J Biol Chem* **270**: 28519-28522.

Díez, E., Alvaro, J., Espeso, E.A., Rainbow, L., Suarez, T., Tilburn, J. *et al.* (2002) Activation of the *Aspergillus* PacC zinc finger transcription factor requires two proteolytic steps. *EMBO J* **21**: 1350-1359.

Dufresne, M., Lespinet, O., Daboussi, M.J., and Hua-Van, A. (2011) Genome-wide comparative analysis of pogo-like transposable elements in different *Fusarium* species. *J Mol Evol* **73**: 230-243.

Espeso, E.A., and Peñalva, M.A. (1994) In vitro binding of the two-finger repressor CreA to several consensus and non-consensus sites at the *ipnA* upstream region is context dependent. *FEBS Lett* **342**: 43-48.

Evangelinos M, Anagnostopoulos G, Karvela-Kalogeraki I, Stathopoulou PM, Scazzocchio C, Diallinas G. (2015) Minos as a novel Tc1/mariner-type transposable element for functional genomic analysis in *Aspergillus nidulans*. *Fungal Genet Biol* **81**:1-11.

Fernández-Martínez, J., Brown, C.V., Díez, E., Tilburn, J., Arst, H.N., Jr., Peñalva,M. A., and Espeso, E.A. (2003) Overlap of nuclear localisation signal and specific DNA-binding residues within the zinc finger domain of PacC. *J Mol Biol* **334**: 667-684.

Hammond, T.M., Andrewski, M.D., Roossinck, M.J., Keller,N.P. (2008a) *Aspergillus* mycoviruses are targets and suppressors of RNA silencing. *Eukaryot Cell* **7:** 350-357

Hammond, T.M., Bok, J.W., Andrewski, M.D., Reyes-Dominguez, Y., Scazzocchio, C., and Keller,N.P. (2008b) RNA silencing gene truncation in the filamentous fungus *Aspergillus nidulans*. *Eukaryot Cell* **7**: 339-349.

Hey, P., Robson,G., Birch, M., and Bromley, M. (2008) Characterisation of Aft1 a Fot1/Pogo type transposon of *Aspergillus fumigatus*. *Fungal Genet Biol* **45**: 117-126.

Izsvak, Z., Chuah, M.K., Vandendriessche, T., and Ivics, Z. (2009) Efficient stable gene transfer into human cells by the Sleeping Beauty transposon vectors. *Methods* **49**: 287-297.

Kupfer, D.M., Reece, C.A., Clifton, S.W., Roe, B.A., and Prade, R.A. (1997) Multicellular ascomycetous fungal genomes contain more than 8000 genes. *Fungal Genet Biol* **21**: 364-372.

Li Destri Nicosia, M.G., Brocard-Masson, C., Demais, S., Hua, V.A., Daboussi, M.J., and Scazzocchio, C. (2001) Heterologous transposition in *Aspergillus nidulans*. *Mol Microbiol* **39**: 1330-1344.

Muñoz-Lopez, M., and García-Pérez, J.L. (2010) DNA transposons: nature and applications in genomics. *Curr Genomics* **11**: 115-128.

Nyyssönen, E., Amutan, M., Enfield, L., Stubbs, J., and Dunn-Coleman, N.S. (1996) The transposable element Tan1 of *Aspergillus niger* var. *awamori*, a new member of the Fot1 family. *Mol Gen Genet* **253**: 50-56.

Orejas, M., Espeso, E.A., Tilburn, J., Sarkar, S., Arst, H.N., Jr., and Peñalva, M.A. (1995) Activation of the *Aspergillus* PacC transcription factor in response to alkaline ambient pH requires proteolysis of the carboxy-terminal moiety. *Genes Dev* **9**: 1622-1632.

Peñalva, M.A. (2005) Tracing the endocytic pathway of *Aspergillus nidulans* with FM4-64. *Fungal Genet Biol* **42**: 963-975.

Peñas, M.M., Hervás-Aguilar, A., Munéra-Huertas, T., Reoyo, E., Peñalva, M.A., Arst, H.N., Jr., and Tilburn, J. (2007) Further characterization of the signaling proteolysis step in the *Aspergillus nidulans* pH signal transduction pathway. *Eukaryot Cell* **6**: 960-970.

Perez-Esteban, B., Orejas, M., Gomez-Pardo, E., and Peñalva, M.A. (1993) Molecular characterization of a fungal secondary metabolism promoter: transcription of the *Aspergillus nidulans* isopenicillin N synthetase gene is modulated by upstream negative elements. *Mol Microbiol* **9**: 881-895.

Rubin, E., and Levy, A.A. (1997) Abortive gap repair: underlying mechanism for Ds element formation. *Mol Cell Biol* **17**: 6294-6302.

Sibthorp, C., Wu, H., Cowley, G., Wong, P.W., Palaima, P., Morozov, I.Y. *et al.* (2013) Transcriptome analysis of the filamentous fungus *Aspergillus nidulans* directed to the global identification of promoters. *BMC Genomics* **14**: 847.

Skipper, K.A., Andersen, P.R., Sharma, N., and Mikkelsen, J.G. (2013) DNA transposon-based gene vehicles - scenes from an evolutionary drive. *J Biomed Sci* **20**: 92.

Tilburn,J.; Scazzocchio,C.; Taylor,G.G.; Zabicky-Zissman,J.H.; Lockington,R.A.; Davies,R.W.(1983) Transformation by integration in *Aspergillus nidulans*. *Gene* **26**: 205-221.

Tilburn, J., Sarkar, S., Widdick, D.A., Espeso, E.A., Orejas, M., Mungroo, J. *et al.* (1995) The *Aspergillus* PacC zinc finger transcription factor mediates regulation of both acid- and alkaline-expressed genes by ambient pH. *EMBO J* **14**: 779-790.

Timakov, B., Liu, X., Turgut, I., and Zhang, P. (2002) Timing and targeting of P-element local transposition in the male germline cells of *Drosophila melanogaster*. *Genetics* **160**: 1011-1022.

Tower, J., Karpen, G.H., Craig, N., and Spradling, A.C. (1993) Preferential transposition of *Drosophila* P elements to nearby chromosomal sites. *Genetics* **133**: 347-359.

Wicker, T., Yahiaoui, N., and Keller, B. (2007) Illegitimate recombination is a major evolutionary mechanism for initiating size variation in plant resistance genes. *Plant J* **51**: 631-641.
